# SmartScanPCOS: A feature-driven approach to cutting-edge prediction of Polycystic Ovary Syndrome using Machine Learning and Explainable Artificial Intelligence

**DOI:** 10.1016/j.heliyon.2024.e39205

**Published:** 2024-10-11

**Authors:** Umaa Mahesswari G, Uma Maheswari P

**Affiliations:** Department of Computer Science and Engineering, College of Engineering Guindy, Anna University, Chennai, 600025, Tamil Nadu, India

**Keywords:** Polycystic ovarian syndrome (PCOS), Explainable artificial intelligence, eXplainable artificial intelligence (XAI), Machine learning, Classification, Ensemble model, Cross validation, Health care

## Abstract

PolyCystic Ovarian Syndrome (PCOS) poses significant challenges to women's reproductive health due to its diagnostic complexity arising from a variety of symptoms, including hirsutism, anovulation, pain, obesity, hyperandrogenism, and oligomenorrhea, necessitating multiple clinical tests. Leveraging Artificial Intelligence (AI) in healthcare offers several benefits that can significantly impact patient care, streamline operations, and improve medical outcomes overall. This study presents an Explainable Artificial Intelligence (XAI)-driven PCOS smart predictor, structured as a hierarchical ensemble consisting of two tiers of Random Forest classifiers following extensive analysis of seven conventional classifiers and two additional stacking ensemble classifiers. An open-source data set comprising numerical parametric features linked to PCOS for classifier training was used. Moreover, to identify essential features for PCOS prediction three feature selection methods: Threshold-driven Optimized Principal Component Analysis (TOPCA), Optimized Salp Swarm (OSSM), and Threshold-driven Optimized Mutual Information Method (TOMIM) were fine-tuned through thresholding and improvisation to detect diverse attribute sets with varying numbers and combinations. Notably, the two-level Random Forest classifier model outperformed others with a remarkable 99.31 % accuracy by employing the top 17 features selected through the Threshold-driven Optimized Mutual Information Method (TOMIM) along with anoverallaccuracy of 99.32 % with 8 fold cross validation for 25 runs. The Smart predictor, constructed using Shapash - a Python library for Explainable Artificial Intelligence - was utilized to deploy the two-level Random Forest classifier model. Ensuring transparency and result reliability, visualizations from robust Explainable AI libraries were employed at different prediction stages for all considered classifiers in this study.

## Highlights

• Fine-tuned feature selection techniques boost the performance of the proposed ensemble classifier.
• The PCOS Smart Predictor integrates Ensemble Machine Learning with Explainable AI for feature contribution insights.
• The model provides valuable guidance to medical experts by revealing key feature influences in PCOS pediction.
• The system supports advanced PCOS analysis in remote areas with growing healthcare facilities.

## Graphical abstract

**Figure:**
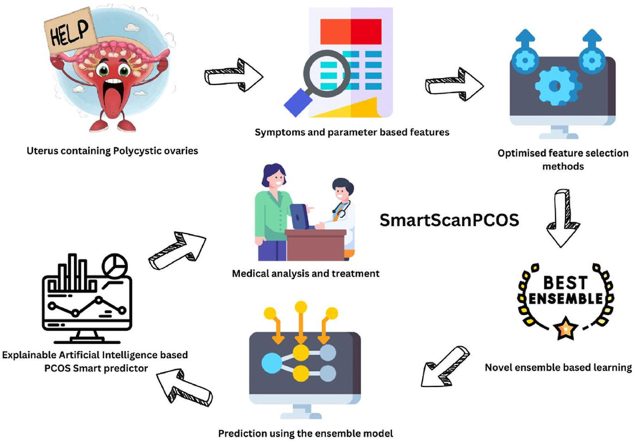
Overview of SmartScanPCOS methodology.

## 1 Introduction

The well-being of women is fundamental to the welfare of families and the advancement of a society. In recent times, shifts in lifestyle, dietary patterns, environmental factors, and societal expectations have notably influenced the deterioration of women's ovarian health [1,2]. The emergence of fluid-filled sacs in the ovaries is a hallmark of Polycystic Ovarian Syndrome (PCOS), associated with various unestablished health risks [3].

According to the Rashtriya Kishor SwathyaKaryakram study [4], around 18 % of women in India, predominantly from the Eastern regions, experience this syndrome, with approximately 70 % of cases remaining undiagnosed and according to data from the World Health Organization (WHO), approximately 116 million women worldwide, accounting for 3.4 % of the global female population, are impacted by PCOS [5].

This condition, prevalent among women in different age groups, poses a significant threat to both their physical and mental health. Characterized by heightened androgen levels, weight fluctuations, irregular menstruation, excessive body hair, hair loss, and male-pattern baldness, PCOS presents a range of concerning symptoms [6].Timely and precise identification of cysts is crucial for swift recovery from this ailment. Numeric parameters, encompassing details about hormone levels and lifestyle, hold significant importance in the evaluation and detection of Polycystic Ovarian Syndrome [7].

The diagnostic process for PCOS commonly includes reviewing medical history, conducting a physical examination, administering blood tests, performing ultrasound imaging, and ruling out other potential conditions which could be time and energy consuming. These diagnostic procedures could also introduce human error, potentially leading to inaccurate diagnoses. Ensuring the precision of PCOS detection and, more broadly, the identification of any healthcare-related condition, holds utmost significance. Accurate detection forms the cornerstone for effective and timely interventions, empowering healthcare providers to devise tailored treatment plans [8].

In the intricate landscape of PCOS, characterized by diverse symptoms, precise detection is paramount to comprehend its multifaceted nature and customize interventions for individual patient requirements. False positives or negatives could result in improper treatments, unnecessary anxiety, or delayed interventions.

Moreover, precise detection facilitates early diagnosis [9], enhancing the management of the condition and potentially averting associated complications. The incorporation of advanced technologies, especially Machine Learning and Artificial Intelligence [10], further elevates the accuracy and efficiency of disease detection, revolutionizing healthcare practices. As the healthcare sector increasingly embraces these technologies, ensuring the dependability and precision of detection outcomes becomes imperative for optimizing patient care.

Furthermore, eXplainable Artificial Intelligence (XAI) aims to improve human understanding and accessibility to AI decision-making processes [11]. In contemporary healthcare initiatives, explainability is key to ensuring robustness in models. For instance, in a pioneering brain tumor detection system using convolutional neural networks on MRI slices, the incorporation of model explainability clarified the network's decision-making process, boosted transparency, identified biases and aided in selecting suitable training data [12].

Furthermore, explainability and feature reproducibility were incorporated into a deep learning-based method for COVID-19 detection [13]. Utilizing the Grad-CAM algorithm, the study scrutinized the alignment of suggested lung X-ray areas with the classification results to validate the system's findings.

Moreover, for the prediction of coronary artery disease, a reliable system was developed, consisting of multimodal predictive models that integrated clinical and radiomic features. These models employed explainable classifiers and interpretable features to aid in clinical decision-making processes [14]. Therefore, it is apparent that the usage of eXplainable Artificial Intelligence (XAI) in healthcare applications contributes to creating trust in AI systems thereby enabling better comprehension of model predictions.

Existing PCOS prediction systems integrate a diverse array of traditional and ensemble classifiers alongside feature selectors. However, there is limited exploration into the potential of incorporating explainability into models handling numerical parametric data. For example, one study advocated for the use of 28 features selected through the Random Forest embedded feature selection method on the same dataset utilized in this study, employing a random forest classifier for classification [15].

Another study utilized two sets of data from follicular fluid and plasma samples for PCOS screening using a stacking classifier [16]. Only a handful of studies like in Ref. [17] investigate the feasibility of employing local and global modal explainability for PCOS detection. In the scarcity of such robust, interpretable machine learning solutions for precise PCOS prediction lies the motivation behind this study.

Combining the interpretive strength of XAI with the precise predictive capabilities of machine learning models could enhance both the accuracy and interpretability of PCOS prediction systems, streamlining the process and reducing reliance on extensive expert clinical tests and resources. Such systems hold the potential to reach women in rural and inaccessible areas, preventing cases from going unnoticed or untreated.

Hence, this research aims to integrate and explore XAI techniques with traditional and ensemble machine learning classifiers to predict PCOS. Additionally, it proposes a best performing ensemble machine learning classifier that employs the minimal and optimal amount of prioritized features for more efficiently detecting PCOS through patient's symptoms and test results dataset. The important contributions made to achieve the goals of this research work are described below.

• To identify key features essential for PCOS prediction, three distinct feature selection techniques (Principal Component Analysis, Salp Swarm Optimization, Mutual Information) were optimized using threshold-driven and fine-tuning approaches. Each technique prioritized features using its unique method, choosing diverse collections of features with varying properties from a dataset. These selected features were then utilized in machine learning classifiers for PCOS detection.
• A two level random forest ensemble classifier with a hierarchical sequence of two levels of random forest classifiers to categorize the dataset into groups of individuals with PCOS and those without PCOS.
• Eleven other classifiers were used in order to determine the efficacy of the proposed classifier. These included two different stacking ensemble models with diverse base learners ensemble using stacking method with different final estimators and nine traditional classifiers (Logistic Regression, Support Vector Machines, Decision Tree, Random Forest, Naive Bayes, K-Nearest Neighbours, AdaBoost, XGBoost, and Extra Trees). Subsequently, a detailed comparative performance analysis was conducted, evaluating the traditional classifiers, the two types of stacking ensemble models, and the proposed ensemble model across various performance parameters using feature sets obtained from the chosen feature selection technique.
• To construct an XAI driven Smart Predictor for PCOS prediction, utilizing the predictions from the proposed two-level random forest classifier. Additionally, implementing diverse XAI-based interpretations at different levels for the predictions generated by other classifiers considered.

The article's remaining sections are organized as follows: The background study is presented in Section 2; the materials and methodology used in this study are explained in Section 3; the result analysis and comparative findings are discussed in Section 4; and finally, Sections 5, 6 contain a discussion and conclusion that highlights the main findings of the study along with a comparison to earlier research, advantages, limitations, and future goals.

## 2 Related work

Polycystic Ovary Syndrome (PCOS) is associated with a range of health complications, with symptoms that markedly differ from those in women who ovulate regularly. These symptoms include increased thickness of the endometrium, insulin resistance, abnormal lipid levels, hypertension, cardiovascular issues, and type-2 diabetes [[18], [19], [20]].

Hormonal disruptions, such as elevated follicle-stimulating hormone (FSH), anti-mullerian hormone (AMH), and luteinizing hormone (LH), are also commonly observed in individuals with PCOS [21,22].

Other signs strongly linked to PCOS are excessive body or facial hair, accelerated hair loss, darkened skin patches, higher body mass index (BMI), obesity, and a tendency for abdominal obesity, which results in a larger waist-to-hip ratio. Poor dietary habits, including frequent fast food consumption, further contribute to the condition [[23], [24], [25]].

Due to the broad impact of PCOS on health, timely and precise diagnosis is essential, and the expertise of healthcare providers plays a vital role in ensuring the accuracy of these diagnoses [26]. The complexity of PCOS and its various manifestations have fueled interest in computer-aided diagnostic systems, which offer the potential for quicker, more precise diagnoses with fewer errors and reduced reliance on human labor [27].

Machine learning (ML) techniques have emerged as essential tools for analyzing the vast amount of healthcare data produced in modern medical settings. They enable intelligent disease diagnosis by effectively processing large, diverse clinical datasets [28].

A number of studies have applied ML to detect PCOS based on patient symptoms. For instance, Aroni Saha Prapthy et al. [29] employed a Random Forest decision tree approach that achieved 93.5 % accuracy using multi-voting, surpassing the performance of K-Nearest Neighbour (KNN), Support Vector Machine (SVM), and Naive Bayes classifiers. Their research emphasized the significance of features such as cycle length, BMI, and follicle count. In a similar vein, Ashwini Kodipalli et al. [30] utilized a Fuzzy TOPSIS method alongside SVM to predict PCOS and related mental health issues, attaining notable accuracy levels of 98.2 % and 94.01 %, respectively. Shazia Nasim et al. [31] introduced the CS-PCOS feature selection technique, which is based on an optimized chi-squared mechanism, and demonstrated that the Gaussian Naive Bayes classifier achieved perfect recall, precision, and F1 scores across multiple metrics. Dana Hdaib et al. [32] developed a diagnostic model using MATLAB and seven classifiers, with the KNN classifier showing superior sensitivity and the Linear Discriminant classifier providing the highest overall accuracy.Meanwhile, Yasmine A. Abu Adla [33] applied a hybrid feature selection technique on a Kaggle dataset and found that the Linear SVM model delivered the best performance, achieving 91.6 % accuracy.

Although significant progress has been made, the literature provides limited coverage on the importance of explainability in PCOS prediction models, a gap that this research seeks to fill. While accuracy, sensitivity, and precision are commonly highlighted, few studies investigate how interpretable machine learning models could improve clinical decision-making. The reliance on black-box models, such as Random Forest and SVM, poses challenges regarding the transparency of their predictions. This is particularly concerning in healthcare, where understanding the rationale behind a diagnosis is just as vital as the result itself.

For example, while Aroni Saha Prapthy et al. [29] and others focus on enhancing classification accuracy, they fail to provide detailed discussions on how these models could be made more interpretable for medical professionals. Similarly, Nasim et al. [31] obtained exceptional outcomes using the Gaussian Naive Bayes classifier, but their work offers little insight into whether or how the key features that drive predictions were communicated to clinicians.

Hence, this study aims to address the importance of explainability by incorporating explainable AI (XAI) methods, specifically SHAP values, into PCOS detection models. The proposed approach not only delivers strong accuracy but also creates a transparent system that helps clinicians better understand how various features, like hormone levels, BMI, and follicle count, contribute to the diagnostic outcome.

Through the use of a two-level ensemble classifier enhanced with XAI technology [34], the research closes the gap between model performance and interpretability. This model offers clinicians not only reliable diagnostic capabilities but also clear justifications for its predictions, making it more clinically relevant. Compared to existing models, which are often highly accurate but lack the necessary transparency, this approach marks a substantial improvement in balancing interpretability with predictive power.

## 3 Materials and methods

In this study, to gauge classifier effectiveness, a comparative analysis was undertaken, utilizing multiple classification models and feature prioritization techniques. Their performance was assessed through various metrics and the classifiers that exhibited superior classification performance has been put forward. An Explainable Artificial Intelligence based PCOS smart predictor utilizing a two-tiered hierarchical Random Forest ensemble machine learning classifier is presented. The classifier underwent training and testing using feature sets selected by three distinct optimized feature selectors.

**Fig. 1:**
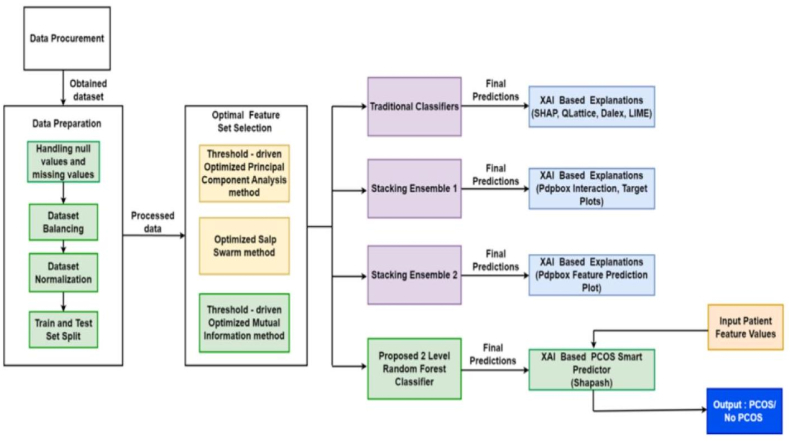
Detailed research framework architecture.

The primary focus is on predicting the presence of PCOS based on symptom data using the designed XAI based smart predictor for the two-tiered Random Forest ensemble using the best performing feature selection method. A detailed architecture of the research framework has been presented in Fig. 1.

This research utilized the open-source real-time PCOS dataset from Kaggle, gathered by Prasoon Kottarathil from patients across 10 hospitals in Kerala. Upon retrieving data from the Kaggle repository, rigorous data processing was employed to refine the dataset, preparing it in a clean and suitable format for machine learning applications.

Following a thorough investigation, three distinct optimized feature selection procedures were used to generate several sets of reduced features, each comprising a different amount of the most significant traits. The study also investigated a variety of machine learning algorithms, including nine classical classifiers and two types of stacking ensemble models. The detailed approaches used in this research are discussed in the following subsections.

### 3.1 Data procurement

The supervised machine learning models were trained using a dataset of patient symptoms and PCOS diagnosis findings from the publicly available 'Kaggle' data collection [35]. The initial review of the PCOS records revealed a total of 541 entries of female patient data, with 44 columns including varied clinical information, including 24 noninvasive measures and 20 invasive parameters, all related to PCOS.

The attributes "Sl. No" and "Patient File No." were omitted from the 44 columns because they had no value in the classification procedure. Among the remaining 42 columns, 'PCOS(Y/N)' was the goal column for training, representing PCOS diagnosis results with 'Yes' and 'No' values. This column's values showed an unequal distribution, with 364 entries suggesting 'No PCOS' and 177 showing 'PCOS'. This data imbalance was dealt with during the preparation process. The set of 41 remaining features encompasses a mix of numerical and categorical attributes relevant to the PCOS condition.

#### 3.1.1 Feature analysis and distribution

To enhance the reproducibility of the study, a comprehensive analysis was conducted on the number of instances and the distribution of features. This meticulous examination provided a clearer understanding of the dataset. Table 1 encapsulated the findings of this analysis, covering 42 features, shedding light on their significance.

**Table 1:** Feature analysis – Number of instances and feature distribution.

S.No.	Feature names	Description
1	PCOS (Y/N)	The target variable included 362 instances labeled as '0' (indicating no PCOS) and 176 instances labeled as '1' (indicating PCOS).
2	Age (yrs)	The most common age was 28 years old (45 instances), followed by 32 years old (44 instances) and 35 years old (37 instances).
3	Weight (Kg)	The weights varied widely, with the most common being 60.0 kg (36 instances) and 50.0 kg (34 instances).
4	Height (*Cm*)	The two most common heights were 152.0 cm (65 occasions) and 158.0 cm (66 instances).
5	BMI	The BMI varied greatly, with 23.11 being the most common result at 10 instances.
6	Blood Group	The distribution displayed 205 instances with the value '15′, 133 with '13′, and 108 with '11'.
7	Pulse rate (bpm)	The most frequent pulse rate is 72 bpm (271 occasions), followed by 74 bpm (100 instances).
8	RR (breaths/min)	The respiratory rate in the majority of cases is between 18 and 20 breaths per minute (271 and 183, respectively).
9	Hb (g/dl)	Haemoglobin levels varied, with the most common values being 11.00 g/dl (61 occurrences) and 10.80 g/dl.
10	Cycle (R/I)	389 instances were regular (0), and 149 were irregular (1).
11	Cycle length (days)	The most frequent cycle lengths were 5 days (275 instances) and 6 days (91 instances).
12	Marriage Status (Yrs)	The distribution included various durations, with the highest being 4.0 years (52 instances) and 3.0 years (51 instances).
13	Pregnant (Y/N)	332 instances were '0' (No) and 206 were '1' (Yes).
14	No. of abortions	434 instances had '0′ abortions, 69 had '1′, and 22 had '2'.
15	I beta-HCG (mIU/mL)	The most frequent value of I beta-HCG (mIU/mL) was 1.99 mIU/mL, with 190 instances.
16	II beta-HCG (mIU/mL)	Similarly, the most frequent value of II beta-HCG (mIU/mL) was 1.99 mIU/mL, with 305 instances.
17	FSH (mIU/mL)	The distribution of FSH (mIU/mL) was highly varied, with the most frequent value being 5.710 mIU/mL (6 instances).
18	LH (mIU/mL)	The distribution of LH (mIU/mL) included many unique values, with the most frequent being 0.20 mIU/mL (5 instances).
19	FSH/LH	The ratio distribution of FSH/LH showed 2.83 as the most common value (6 instances).
20	Hip (inch)	The most common hip measurements were 38 inches (106 instances) and 36 inches (65 instances).
21	Waist (inch)	The most frequent waist measurements were 34 inches and 32 inches, each with 85 instances.
22	WaistHip Ratio	The most common waist-to-hip ratio was 0.89 (68 instances).
23	TSH (mIU/L)	The most frequent TSH (mIU/L) value was 5.000 mIU/L (18 instances).
24	AMH (ng/mL)	The distribution of AMH (ng/mL) was diverse, with 1.00 ng/mL and 2.50 ng/mL being the most frequent (9 instances each).
25	PRL (ng/mL)	The values of PRL (ng/mL) were highly varied, with the most frequent being 25.04 ng/mL, 8.10 ng/mL, and 12.11 ng/mL (each with 3 instances).
26	Vit D3 (ng/mL)	The most frequent Vit D3 (ng/mL) value was 18.70 ng/mL (6 instances).
27	PRG (ng/mL)	The most common PRG (ng/mL) value was 0.250 ng/mL (171 instances).
28	RBS (mg/dl)	The most frequent RBS (mg/dl) values were 100.0 mg/dl (111 instances) and 92.0 mg/dl (106 instances).
29	Weight gain(Y/N)	334 instances were labeled '0' (No) for weight gain, and 204 were labeled '1' (Yes).
30	Hair growth(Y/N)	390 instances were labeled '0′ for hair growth, and 148 were labeled '1'.
31	Skin darkening (Y/N)	373 instances were labeled '0′ for skin darkening, and 165 were labeled '1'.
32	Hair loss (Y/N)	294 instances were labeled '0′ for hair loss, and 244 were labeled '1'.
33	Pimples (Y/N)	274 instances were labeled '0′ for pimples, and 264 were labeled '1'.
34	Fast food (Y/N)	278 instances were labeled '1′ for fast food consumption, and 260 were labeled '0'.
35	Reg. Exercise (Y/N)	406 instances were labeled '0′ for regular exercise, and 132 were labeled '1'.
36	BP Systolic (mmHg)	The most frequent systolic blood pressure values were 110 mmHg (264 instances) and 120 mmHg (251 instances).
37	BP Diastolic (mmHg)	The most common diastolic blood pressure values were 80 mmHg (379 instances) and 70 mmHg (157 instances).
38	Follicle No. (L)	The most frequent number of follicles on the left ovary was 3 (61 instances).
39	Follicle No. (R)	The most frequent number of follicles on the right ovary was 5 (55 instances).
40	Avg. F size (L) (mm)	The most frequent average follicle size on the left ovary was 15.0 mm (76 instances).
41	Avg. F size (R) (mm)	The most frequent average follicle size on the right ovary was 18.0 mm (84 instances).
42	Endometrium (mm)	The most frequent endometrial thickness was 9.0 mm (52 instances).

This detailed breakdown captured key patterns and trends thereby providing a deeper comprehension of the variables at play, underscoring their potential impact and relevance in the context of the research.

**Fig. 2:**
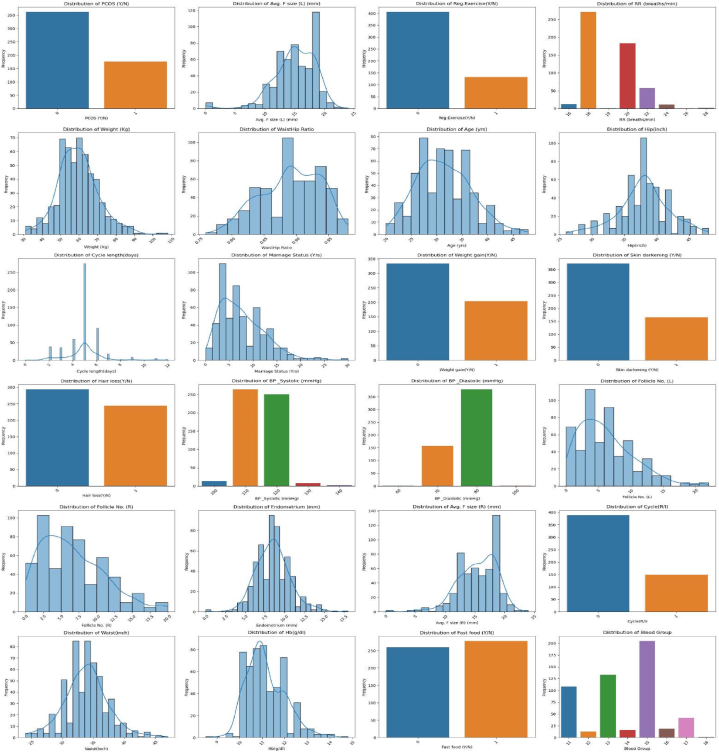
Feature Distribution of significant numerical and categorical features.

Additionally, Fig. 2 presents the distribution of 23 significant features along with the target variable. In this illustration, histograms were utilized to display the distribution of numerical features, while bar plots were employed to visualize the distribution of categorical features. This extensive visualization offered a clear and informative overview, emphasizing the variability and frequency of each feature in relation to the target variable.

### 3.2 Data preparation

In this study, the dataset was meticulously prepared to eliminate any flaws or anomalies, confirming its suitability for use in machine learning models. Several procedures were implemented to address issues such as missing or contradictory data samples, inconsistencies, noise, and other anomalies in the dataset. The sections that follow provide a full summary of the steps performed to prepare the dataset.

Initially, the dataset was examined for the presence of null and missing values, and rows containing null values were removed. Features with a limited number of null values were addressed by replacing the null values with zero. The "BMI", "FSH/LH", and "Waist:Hip Ratio" were all rounded to two decimal places.

Following this, efforts were made to balance the data for training, taking into account the dataset's imbalanced target attribute, which included 364 non-PCOS records and 177 PCOS records. To establish a more equitable distribution, oversampling was performed using the 'Borderline Synthetic Minority Oversampling Technique (SMOTE),' a modification of the conventional SMOTE technique used to cope with imbalanced datasets, particularly in classification difficulties [36]. This approach creates synthetic samples for the minority class to address the imbalance in target attribute values [37].

The SMOTE technique, detailed in Equation (1), comprises producing a synthetic sample ($x_{sample}$) using the minority class value (x) and picking a randomly chosen value $\left(x_{random}\right)$ from the nearest neighbours of x, with 0 ≤ η ≤ 1. Using Borderline SMOTE, the dataset instance count grew to 724, including 362 positive and negative PCOS diagnosis results.

(1) $$Synthetic\;sample\;\left(x_{sample}\right)=x+\eta \times \left(x_{random}-x\right)$$

### 3.3 Optimal feature set selection

The feature selection process [38] is crucial in the machine learning pipeline, enhancing the efficiency, interpretability, and generalizability of models. It involves identifying the optimal subset(s) of features for the most effective data representation. In this study, the pre-processed dataset consists of 41 attributes. Including all of them in the classifier could potentially reduce accuracy.

Therefore, a thorough selection and prioritization of features were carried out using three distinct feature selection techniques. Each of the three feature selection methods-Principal Component Analysis (PCA), Salp Swarm Optimization (SSO), and mutual information-may harbor potential biases.

Due to its sensitivity to feature scaling, reliance on linear correlations, vulnerability to outliers, arbitrary dimensionality reduction choices, and assumption of a normal data distribution, the results of PCA could be biased [39]. The biases in SSO are caused primarily by a lack of resilience, limited exploration, initialization sensitivity, parameter selections, and fitness function dependence [40]. Biases in mutual information include a preference for particular data distributions, a failure to consider feature correlation, sensitivity to sample size, subjective threshold selection, and incapacity to identify non-linear correlations [41]. The validity of results may be impacted by certain biases, which can skew selections.

Considering the biases associated with these methods, all three feature selection techniques were optimized using thresholding and iterative determination in this study to derive the optimal set of unbiased features from the PCOS dataset. The subsequent section elaborates on these techniques.

#### 3.3.1 Threshold-driven optimized Principal Component Analysis method (TOPCA)

The dimensionality of a dataset can be decreased through Principal Component Analysis (PCA) [42]. This is a type of filter method that represents the dataset with a reduced set of essential variables called principal components. The key components are determined by explained variance and play crucial roles in each principal component.

Principal Component Analysis presents several advantages over alternative methods by reducing dimensionality while retaining crucial data features and simplifying complex datasets for easier interpretation and visualization. Furthermore, it efficiently eliminates redundant information and noise, thereby improving the effectiveness of subsequent analyses.

Therefore, in this research, using the PCA function from Scikit-learn, a threshold of 0.95 was established to identify the optimal number of components based on explained variance. The number of components was computed as shown in Equation (3). The function argmax(.) identifies the index of the maximum value within an array of feature indices. In this context, cumulative_variance refers to the array containing cumulative explained variance values for each principal component.

(3) $$\begin{array}{c} \;n-\text{components}=\mathrm{argmax}\left(\;\text{cumulative}\_\text{variance}\geq \text{threshold}\;\right)+1\; \end{array}$$

Out of the 41 original features, 25 were chosen because their cumulative variance exceeded the criteria. Following that, a simple Principal Component Analysis was used to further reduce the dimensionality to 10 features from the resulting set of 25 features, which were then used as input for the classifier.

PRG (ng/mL), average F size (L) (mm), regular exercise (Y/N), I beta - HCG (mIU/mL), RR (breaths /min), weight (kg), waisthip ratio, age (years), hip (inch), and FSH/LH were the features considered

#### 3.3.2 Optimized Salp Swarm method (OSSM)

Salp Swarm Optimization (SSO) is wrapper method inspired by the collective behaviour of marine organisms called salps for optimization [43]. Widely applied in feature selection, SSO excels in systematically navigating the solution space and identifying a relevant set of problem-specific features.

The algorithm emulates the swarm dynamics of salps, where each salp communicates and coordinates with others to uncover an optimal feature set [44]. During optimization, critical feature subsets are represented as positions in the feature space, refined iteratively based on the defined objective function.

Salp Swarm Optimization stands out for its robustness and adaptability in optimization tasks, efficiently exploring solution spaces and exhibiting improved convergence properties. Moreover, it excels in addressing high-dimensional and complex optimization challenges, rendering it applicable across various domains. It also enhances the efficiency and effectiveness of feature selection by collaboratively exploring the solution space.

In this work, the Salp Swarm Optimization algorithm was implemented by defining a fitness function to assess the accuracy of a K-Nearest Neighbours (KNN) classifier using a subset of selected features. The main SSO function initialized Salps and iteratively updated positions, velocities, and evaluates fitness. This process was repeated several times to obtain a stable optimal feature set.

Using the pre-processed dataset, the names of selected features and their best fitness were retrieved aiming to find features maximizing KNN classifier accuracy inspired by Salps collaborative optimization behaviour. After several iterations, the set of coefficients and parameters as in Table 2 were finalized to obtain an optimal SSO based feature selector with best fitness of **91 %** and optimal feature set containing 15 features including: Cycle length(days), Marriage Status (Yrs), FSH(mIU/mL), LH(mIU/mL), WaistHip Ratio, PRG(ng /mL), Weight gain(Y/N), Skin darkening (Y/N), Hair loss(Y/N), Reg.Exercise(Y/N), BP_Systolic (mmHg), BP Diastolic (mmHg), Follicle No. (L), Follicle No. (R), Endometrium (mm).

**Table 2:** Finalized coefficients and parameters.

Coefficients and Parameters	Description	Value
C1	Personal Best Position Update Coefficient	3
C2	Global Best Position Update Coefficient	3
W	Inertia weight	0.9
Max_iter	Maximum number of iterations	100
Population_size	Number of salps in the swarm	30
Num_runs	Number of runs to get a fixed set of optimal features	20

#### 3.3.3 Threshold-driven optimal mutual information method (TOMIM)

Mutual information (MI) is a statistical measure that is of importance in feature selection and evaluates the exchange of information between two variables and significance of features with regards to a target variable [45].

It serves as a measure of uncertainty decrease incurred by a specific feature about the target variable. Because of its ability to imply sturdy relationships and recognize crucial features MI is a widely used feature selection technique that captures intricate, non-linear connections within large datasets [46].

It is proved to improve model interpretability and prediction accuracy by focusing on the pivotal features. Compared to other feature selection methods, Mutual Information offers several distinct advantages. It is model-agnostic, versatile across datasets, nonparametric, capturing complex, non-linear relationships without assuming specific data distributions and straightforward to understand and compute, offering clear measures of feature dependence.

The Mutual Information method for feature selection is scalable, efficiently handling large datasets, evaluates features independently to focus on individual relevance, and is robust to irrelevant features, reducing the risk of being misled by noise. Hence, Mutual Information was adopted in this study for feature selection.

Initially, mutual information scores were calculated between the pre-processed dataset's features and the target variable using the 'mutual_info_classif' function from scikit-learn. Features with scores surpassing the threshold that was fixed to the mean of all the calculated MI scores were chosen for further analysis. This is represented in Equation (4), where mi_scores [i] represents the i- th element in the array of mutual information scores, and the notation {i∣ mi_scores [i]> threshold }represents the indices where the specified condition holds true.

(4) $$\begin{array}{c} \;\text{features}\_\text{selected}=\left\{i|\text{mi}\_\text{scores}\;\left[i\right]>\;\text{threshold}\;\right\}\; \end{array}$$

**Fig. 3:**
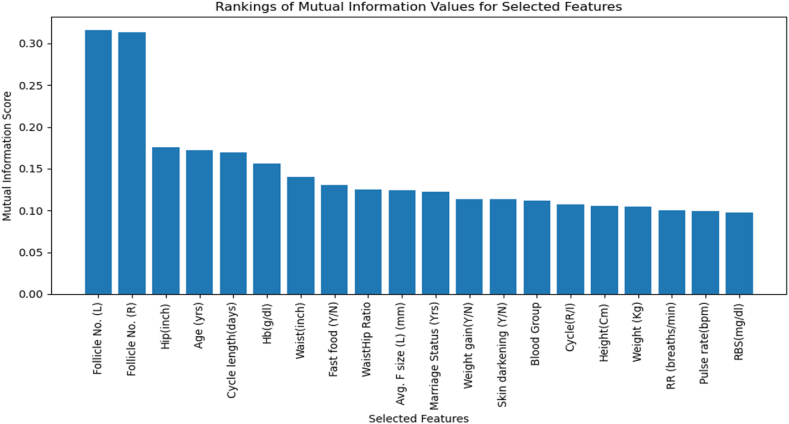
Features selection and ranking by the proposed Threshold-driven Optimal Mutual Information method.

This selected subset of features was employed to train a K-Nearest Neighbours Classifier with 3 neighbours. The trained model predicted the validation set, and its accuracy was computed to be 91 %. Fig. 3 displays the rankings of mutual information values for 17 chosen features, with taller bars representing more informative attributes.

The TOMIM (Threshold-Driven Optimal Mutual Information Method) algorithm pinpointed Follicle No. (L) and Follicle No. (R) as the most crucial features, underscoring their importance in predicting PCOS (Polycystic Ovary Syndrome). Other significant features include Hip (inch), Age (yrs), Cycle length (days), and Hb (g/dl), all contributing substantial information to the model. Mid-ranked features like Waist (inch), Fast food (Y/N), Waist/Hip Ratio, Avg. F size (L) (mm), Marriage Status (yrs), Weight gain (Y/N), and Skin darkening (Y/N) also play important roles, while Blood Group, Cycle (R/Y), Height (cm), Weight (kg), RR (breaths/min), Pulse rate (bpm), and RBS (mg/dl) add further relevance.

The TOMIM approach outperformed other feature selection methods, demonstrating superior model accuracy with a minimal set of features. This selection was corroborated through expert consultations and a comprehensive literature review, confirming the clinical relevance of the features in predicting PCOS.

### 3.4 Machine learning models and explainable AI

In machine learning, classification is the building of models that learn from training data to predict the class labels of samples. This acquired knowledge is then applied to classify new input data into predefined class labels that were established during the training process [47].

This study employs binary classification, where instances are categorized into either the "PCOS" or "No PCOS" class based on the training set using 4 classification techniques (traditional classifiers, stacking ensemble one, stacking ensemble two, two level random forest classifier).

Each of the models underwent training, testing, and evaluation using the set of features selected by the feature selectors. All the models were configured to leverage GPU acceleration through the utilization of NVIDIA, CUDA, and a TensorFlow based GPU setup. The predictions of the classifiers were visualized using various explainable artificial intelligence techniques. The techniques are detailed below.

#### 3.4.1 Traditional machine learning classifiers and explainable artificial intelligence

Traditional machine learning classifiers are highly sought after in the realm of supervised learning due to their clarity and transparency. These algorithms leverage labeled training data to discern patterns in input data and the corresponding features linked to output labels.

**Fig. 4:**
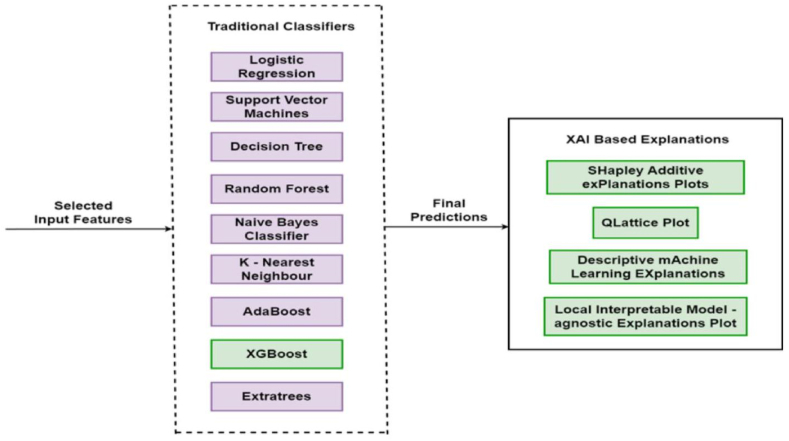
Architecture of the chosen Traditional classifiers and XAI based Explanation methods.

The goal is to construct models capable of accurately predicting labels for unseen data. The study used threshold-driven optimal mutual information (TOMIM) to select features for classifiers such as Logistic Regression, Support Vector Machines, Decision Trees, Random Forests, Naive Bayes, K-Nearest Neighbours, Adaboost, XGBoost, and Extra Trees, as shown in Fig. 4.

GridSearchCV is a powerful tool in the Scikitlearn library that conducts an exhaustive search over a predefined parameter grid for an estimator. By evaluating all possible parameter combinations through cross-validation, it identifies the optimal hyperparameters for enhancing model performance [48]. In this research, each classifier underwent hyperparameter tuning using GridSearchCV with 80:20 and 70:30 train-test data split combinations, to identify the optimal set of hyperparameters as in Table 3. Remarkably, XGBoost with a split of 70:30, outperformed it's counterparts, achieving an impressive accuracy of **93 %.**

**Table 3:** Hyperparameters for XGBoost determined by GridSearchCV.

colsample_bytree	gamma	learning_rate	max_depth	min_child_weight
0.5	0.3	0.05	5	1

To improve the transparency and interpretability of the model, this research incorporated Explainable Artificial Intelligence (XAI) techniques. Plots were obtained based on the probabilities of positive class (Presence of PCOS) from the model's predicted probabilities using a lambda function before passing the predictions to the corresponding explainers.

**Fig. 5:**
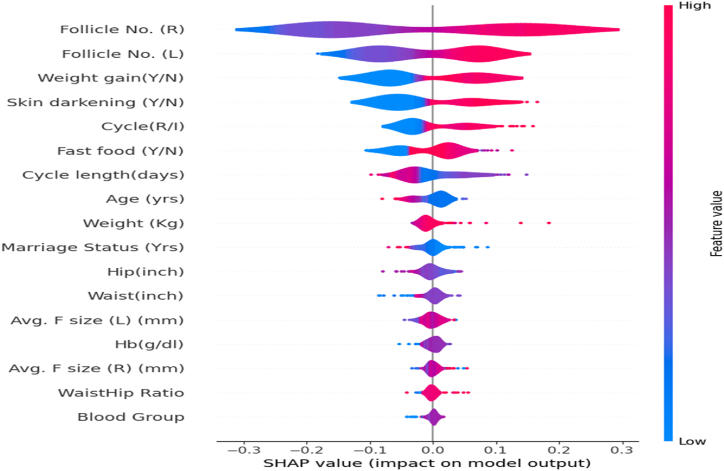
SHAP violin plot.

The Shapley Additive exPlanations explainer as in Fig. 5 constructed a summary plot encompassing all instances within the test data. This reflected the influence of each feature on model predictions based on the estimated SHAP values. The SHAP explainer assigned Shapley values to each feature based on the context of individual predictions made by the model [49]. The x-axis in Fig. 5 represents SHAP values for the respective features, and the color indicates variations in data values, distinguishing between higher and lower values for individual features. A feature with a higher SHAP value, as shown by a broader violin plot, has a bigger impact on the model's output forthat specific prediction.

A positive SHAP value raises the anticipated value, and a negative SHAP value lowers it. Fig. 5 depicts the SHAP values, highlighting that Follicle No. (R) and Follicle No. (L) are the most influential predictors in identifying PCOS, with notable positive and negative effects on the model's output. This underscores the importance of the number of fluid-filled sacs in either ovary as key indicators of PCOS.

Furthermore, non-invasive factors such as weight gain, skin darkening, irregular menstrual cycles, and fast food consumption are also significant contributors to the prediction. The SHAP plot effectively illustrates the significance of these features, with the color gradient bar indicating feature values from low (blue) to high (red), reinforcing their importance in the model. This detailed visualization highlights the multifactorial nature of PCOS and the substantial impact of each feature on the predictive model.

**Fig. 6:**
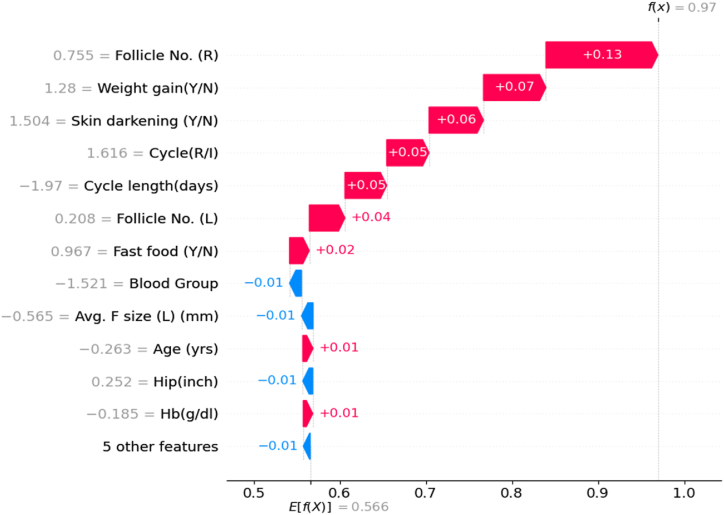
SHAP Waterfall plot.

The SHAP Waterfall plot in Fig. 6 uses expected values on the x-axis to identify the key features influencing PCOS prediction. Each movement in the plot indicates whether a feature positively or negatively impacts the likelihood of a PCOS diagnosis. This plot, based on a randomly selected instance from the test set that is PCOS positive, visually illustrates the significance of features like follicle counts in the ovaries, weight gain, skin darkening, and menstrual irregularities.

The plot's consistency with previous results, such as those in the violin plot, emphasizes the essential role of both follicle-related and non-invasive features in PCOS detection. The terms f(x) and E[f(x)] represent the predicted and expected values, respectively, underscoring the critical importance of these factors in the predictive model.

The QLattice tool plays a pivotal role in Explainable AI by automatically generating a predictive model. In this study, the pre-processed dataset was utilized as input to run a predictive model using QLattice, resulting in a Qgraph that establishes connections between significant input features and the output feature. The model employs initial random weights that undergo optimization throughout the training process.

**Fig. 7:**
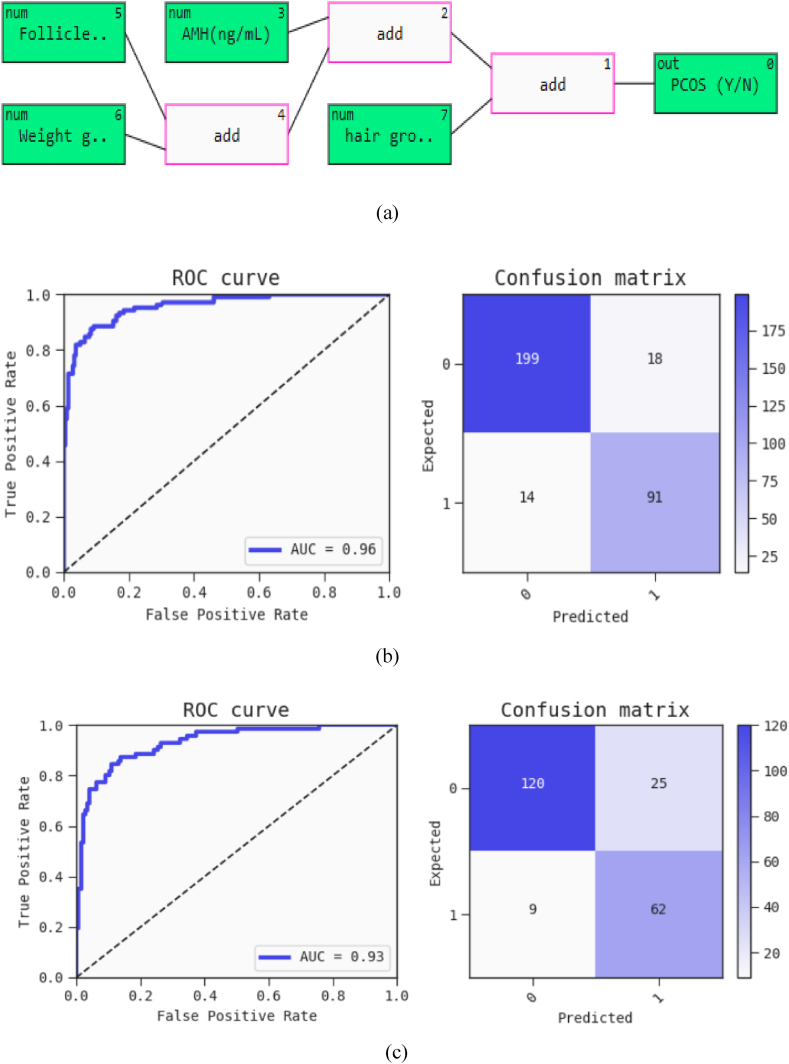
(a) QLattice plot (b) Training ROC curve and confusion matrix (c) Testing ROC curve and confusion matrix of the generated predictive model.

Fig. 7 showcases a QLattice plot that identifies Follicle No. (R), AMH (ng/mL), Weight Gain (Y/N), and Hair Growth as the most significant predictors for PCOS. The QLattice diagram in Fig. 7(a), depicted with white boxes, demonstrates how these key features interact to form a function that predicts the likelihood of PCOS. The consistency of these features with those highlighted in other analyses underscores the reliability of the predictive model, emphasizing the effectiveness of Explainable Artificial Intelligence in pinpointing crucial factors.

The model achieved a training accuracy of 90.1 % and a testing accuracy of 84.3 %, as illustrated by the ROC curves and confusion matrices in Fig. 7(b) and (c) for the respective datasets. This detailed visualization highlights the model's strength and dependability in accurately predicting PCOS.

**Fig. 8:**
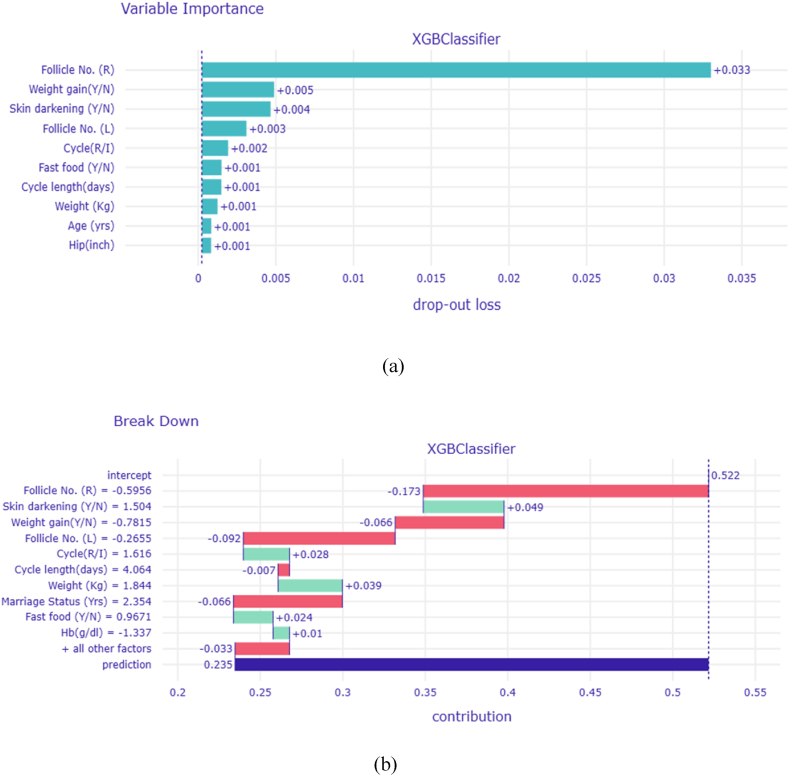
XGBoost Model (a) Feature Variable Importance Plot (b) Break Down Plot for the chosen local explanation.

DALEX is a R package designed to furnish Descriptive mAchine Learning EXplanations, enabling the generation of both local and global explanations for the predictions of a model. In this research, DALEX was utilized to offer an in-depth explanation for a single instance where the XGBoost classifier predicted a positive class, as demonstrated in Fig. 8.

In Fig. 8(a), it is evident that features such as Follicle No. (R), Weight Gain (Y/N), Skin Darkening (Y/N), and Follicle No. (L) hold the highest importance for this particular prediction. Fig. 8(b) presents a breakdown plot that further elucidates the contribution of each feature to the overall prediction.

The intercept row reflects the average prediction value (0.522), while the subsequent rows show how this mean shifts when each feature is adjusted individually. Positive impacts are indicated by green bars, whereas negative effects are marked in red. The final row, labeled "prediction", integrates the mean value with the individual feature contributions to present the predicted probability of PCOS for the given instance, highlighted by the blue bar. This detailed analysis emphasizes the role of each feature in the prediction, offering a transparent and insightful interpretation of the model's decision making process.

Local Interpretable Model-agnostic Explanations (LIME) allows to locally comprehend a learning model by examining the interactions between its internal components. LIME typically generates a collection of explanations that elucidate each feature's contribution to the model's prediction for a given set of input samples.

In this study, LIME was employed to generate a local explanation for a singleinstance (positive case) using the predictions of an XGBoost classifier, which was trained with mutual information. The LIME analysis detailed how various features influenced the classifier's decision, with an intercept value of 0.914 and a prediction score of 1.0238 for the instance, indicating a positive diagnosis of PCOS.

**Fig. 9:**
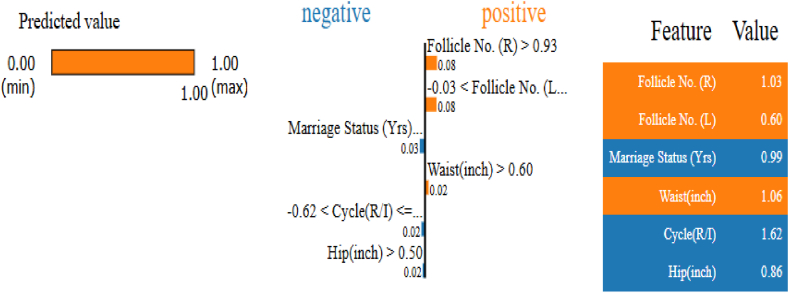
LIME plot for PCOS - positive case instance.

Fig. 9 illustrates that the predicted value of 1 confirms a PCOS diagnosis for the patient. Significant features such as Follicle No. (R), Follicle No. (L), Weight (Kg), and Waist (inch) were shown to positively affect the prediction, whereas Marriage Status (Yrs) and Age (yrs) had a detrimental impact. The feature contributions were: Follicle No. (R) > 0.93 contributed positively by 0.088, with ranges −0.03 < Follicle No.≤0.91 adding 0.086; Marriage Status (Yrs) > 0.48 and −0.08 < Age (yrs)≤0.66 reduced the prediction by −0.033 and −0.024, respectively; Skin Darkening (Y/N) in the range −0.67 to 1.50 added 0.023, and Hip (inch) > 0.50 decreased it by −0.018. This detailed breakdown underscores the significance of each feature in influencing the model's prediction, offering clear insights into the elements driving the PCOS diagnosis.

#### 3.4.2 Stacking ensemble models and explainable artificial intelligence

In the relentless pursuit of advancing predictive modeling beyond the confines of existing methodologies, this research has embarked on a comprehensive exploration of stacking ensemble methods [50]. Stacking, a potent ensemble learning technique in machine learning, orchestrates the collaboration of diverse models, each trained on distinct subsets of data, to collectively elevate the overall predictive performance.

At the crux of this approach lies the integration of a meta-model, meticulously selected to function as the ultimate model. This meta-model takes the individual predictions from each base model as input and synthesizes a definitive prediction as the output.

**Fig. 10:**
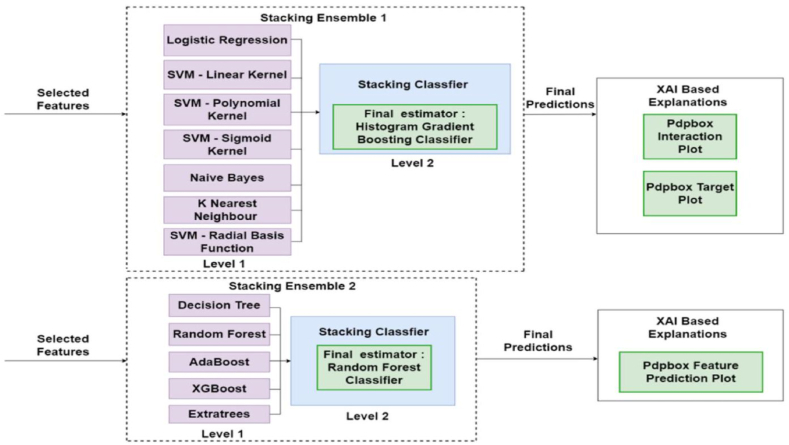
Architecture of the chosen ensembles and XAI based Explanation methods.

As depicted in Fig. 10, this research harnesses the prowess of two distinct stacking ensemble models, and their predictions undergo a thorough explication through two dedicated eXplainable Artificial Intelligence (XAI) techniques. The features singled out by the mutual information module serve as the input to both ensemble models, unraveling the intricacies of their resulting predictions. The inaugural stacking ensemble, denoted as Stacking Ensemble One, employs a sophisticated two-level architecture. Logistic regression, Support Vector Machines with Linear, Polynomial, Sigmoid, and Radial Basis Function kernels, Naive Bayes, and K Nearest Neighbour classifiers are among the seven classifiers integrated at the first level.

On the second level, a Histogram Gradient Boosting Classifier serves as the ultimate estimator, while a Stacking classifier assumes control. Leveraging the features identified through the mutual information method, Stacking Ensemble One attains a commendable accuracy level of **92 %**. The predictions of this ensemble are further scrutinized and expounded upon through the lens of the Partial Dependence Plots (PDP) Toolbox.

Stacking Ensemble Two, mirroring its counterpart, unfolds in a dual-tiered structure. Base classifiers (Decision Tree, Random Forest, AdaBoost, XGBoost, and Extra Trees)work together well in the first level. Comparable to Stacking Ensemble One, the second stage incorporates a Stacking Classifier, with a Random Forest classifier acting as the final arbiter.

With the input features derived from the mutual information method, Stacking Ensemble Two achieves an even higher accuracy rate of **93 %**. The predictions of this ensemble undergo a meticulous explanatory process utilizing the Partial Dependence Plots (PDP) Toolbox. This strategic utilization of stacking ensemble methods coupled with XAI techniques not only amplifies the predictive capabilities of the models but also provides nuanced insights into the complex relationships between the chosen features and the predicted outcomes.

**Fig. 11:**
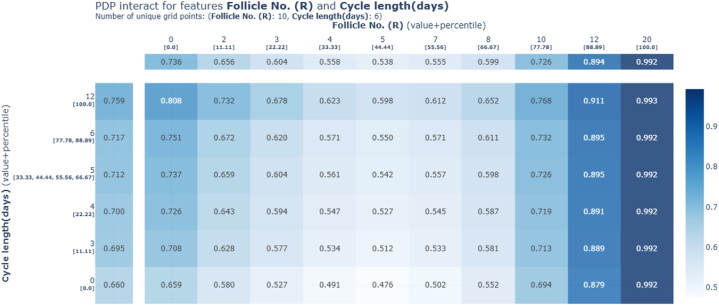
PDP Interaction plot between Follicle No. (R) and Cycle length (days) based on stacking ensemble 1.

**Fig. 12:**
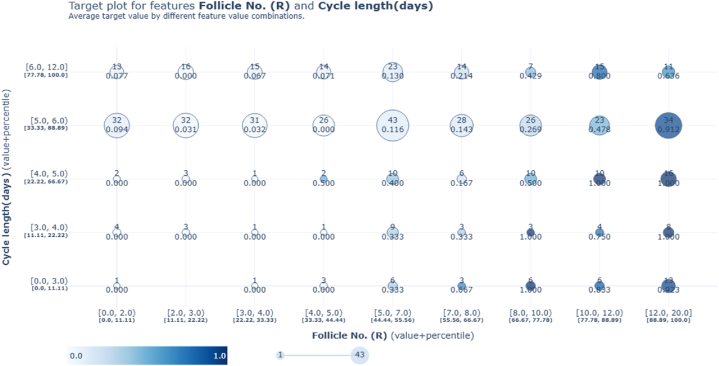
PDP target plot between Follicle No. (R) and Cycle length (days) based on stacking ensemble 1.

Following the application of mutual information, the ensemble stack 1 predictions underwent analysis using the PDP toolkit for explanations. The study involved generating an interaction plot (Fig. 11) and a target plot (Fig. 12) to examine the relationship between Follicle No. (R) and Cycle Length (days).

These visualizations were created to analyze how the number of follicles in the right ovary relates to menstrual cycle duration, particularly within the framework of stacking ensemble 1 predictions. By leveraging percentile values from the ensemble model's outcomes, the plots highlighted a notable effect of follicle count on menstrual cycle length, which in turn improved the model's ability to predict PCOS.

Additionally, the analysis for stacking ensemble 2 focused on the significant feature Follicle No. (R), which was pivotal in earlier PCOS-related findings. The explainer's evaluation of percentile and count values revealed a distinct pattern: as the number of follicles increases, so does the likelihood of PCOS, especially when the count surpasses 6.

**Fig. 13:**
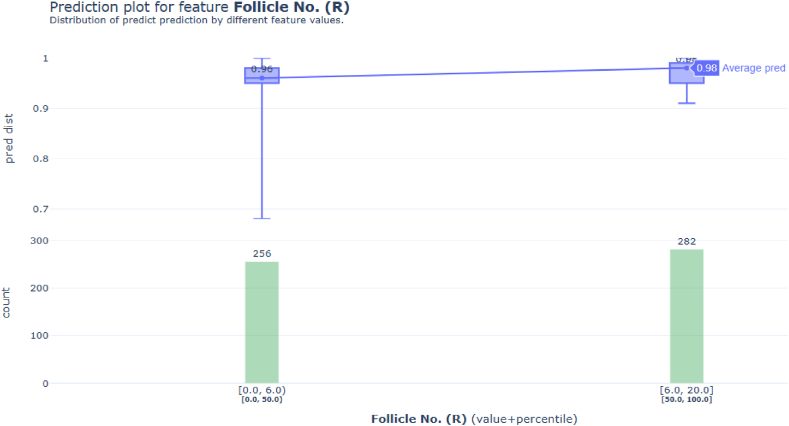
PDP plots for stacking ensemble 2 - Prediction plot for feature Follicle No. (R).

Moreover, Fig. 13 presents a prediction plot that displays the average predicted values for different follicle counts, calculated from the average of predictions (pred_q1, pred_q2, and pred_q3) detailed in Table 4. This graphical representation aids in elucidating how the model reacts to varying follicle counts, further emphasizing the link between a higher follicle count and a greater probability of PCOS.

**Table 4:** Explanation for the prediction.

x	value	percentile	count	pred_q1	pred_q2	pred_q3
0	[0.0,6.0)	[0.0,50.0)	256	0.95	0.96	0.98
1	[6.0,20.0]	[50.0,100.0]	282	0.95	0.98	0.99

#### 3.4.3 XAI based PCOS smart predictor using proposed two level random forest classifier

After exploring the capabilities of the traditional classifiers and the two stacking ensemble models, a two level random forest classifier was developed with superior accuracy and performance compared to the other explored models.

**Fig. 14:**
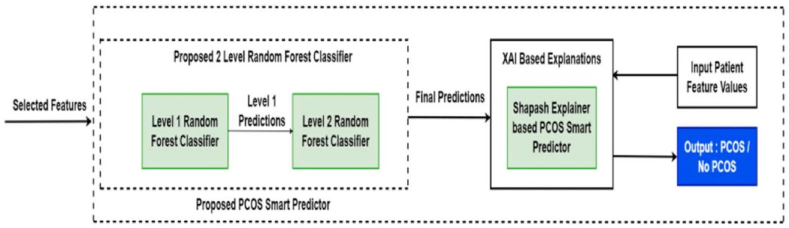
Architecture of the proposed PCOS Smart Predictor.

As depicted in Fig. 14, the 17 features, selected through the optimized mutual information method (TOMIM), were fed into the first-level random forest classifier, comprising 17 estimators. This classifier was trained on the standardized training data, and predictions from the 17 base estimators on the test data were horizontally stacked. Subsequently, these first-level predictions served as features for training the second-level Random Forest Classifier, achieving an accuracy of **99.31 %** on the test data.

In this study, the Smart Explainer class, a key component of the Shapash library was employed to develop a PCOS smart predictor. The predictor was developed with an aim to elucidate predictions made by the 2-level random forest classifier, offering detailed insights into feature contributions and model interpretations based on user-inputted features. The TOMIM-selected reduced feature set, along with the compiled two-level random forest classifier, predictions, and dictionaries containing features and labels, served as inputs to the smart predictor.

Algorithm 1 outlines the workflow using which the PCOS smart predictor was built utilizing the Shapash Python library for eXplainable Artificial Intelligence.

**Algorithm 1:** Prediction using PCOS Smart Predictor.

**Input:** RFTOMIM: Reduced feature set containing 17 features selected by TOMIM. 2LRFC: Compiled Two Level Random Forest Classifier. 2LRFCPred: Predictions of the Two Level Random Forest Classifier. labeldict: Input dictionary of labels to each feature. featuresdict: Input dictionary of features. personx: Input test case of feature values. **Output:**ClassPred: Predicted output class: PCOS/No PCOS Contri: Contribution of individual features to the resultant prediction. 1: xpl ⇐ SmartExplainer(2LRFCPred, labeldict, featuresdict) 2: xpl.compile(2LRFCPred) 3: predictor ⇐ xpl.to_smartpredictor(.) 4: predictor.save(‘./predictor.pkl’) 5: predictor_load ⇐ load_smartpredictor(‘./predictor.pkl’) 6: predictor_load.add_input(personx) 7: **return** ClassPred, Contri

Following a meticulous examination of the dataset with the guidance of medical experts, five sets of test cases were formulated to assess the smart predictor's performance. The outcomes of these tests, as discussed in the subsequent sections, further validate the effectiveness and reliability of the developed PCOS predictor.

Table 5 displays the input feature values for five synthetically generated patient test cases, created to replicate real-world scenarios based on the dataset specifications. Each test case is presented with its corresponding prediction output, probability, and the contributions of individual features to the prediction. These values were meticulously established through thorough consultations with medical experts for this study.

**Table 5:** Prediction and Feature contributions obtained by the Smart Predictor for five test cases (TOMIM).

Selected Features	Test Case 1		Test Case 2		Test Case 3		Test Case 4		Test Case 5	
	Value	Contribution	Value	Contribution	Value	Contribution	Value	Contribution	Value	Contribution
Follicle No. (R)	10	0.010553	5	0.026931	0	0.053373	2	−0.014997	7	0.013797
Marriage Status (Yrs)	2	0.050762	0	−0.070042	2	−0.073793	6	−0.07026	4	0.065465
Follicle No. (L)	7	0.015817	4	0.015438	1	−0.032467	1	−0.031062	15	0.031531
Skin darkening (Y/N)	0	−0.156572	0	−0.152003	0	0.171862	0	0.144626	0	−0.125378
Cycle length (days)	5	0.071869	5	0.096621	4	−0.066037	6	−0.073895	5	0.082493
Avg. F size (L) (mm)	6	0.031168	4	0.066697	0	0.038992	0	0.036115	17	0.058667
Hip (inch)	35	0.091081	32	0.081218	30	−0.054068	39	−0.051074	39	0.081471
Blood Group	11	0.043892	13	0.039742	15	−0.034123	15	−0.044873	15	0.039493
Avg. F size (R) (mm)	9	0.015676	5	0.017256	1	−0.027471	19	−0.027726	20	0.039761
WaistHip Ratio	0.9	0.001439	0.87	0.001759	0.98	0.000468	0.98	−0.000112	0.9	0.000504
Waist (inch)	30	−0.065842	28	−0.068105	24	0.049511	34	0.049234	35	−0.062855
Cycle (R/I)	1	0.002526	1	0.005847	0	0.010082	0	0.009751	0	−0.013744
Hb (g/dl)	10	0.020021	7	0.020407	10	−0.009835	11.2	−0.020634	10	0.025146
\Weight (Kg)	60	0.012733	53	0.004355	43	−0.011265	64	−0.011372	71	0.015751
Age (yrs)	25	0.035211	22	0.028646	27	−0.021086	25	−0.014787	20	0.019608
Weight gain (Y/N)	1	0.054673	1	0.061566	0	0.113558	0	0.113408	0	−0.093407
Fast food (Y/N)	1	−0.002149	1	−0.002296	0	0.012378	0	0.01009	0	−0.004267
Medical expert review	PCOS		PCOS		Not PCOS		Not PCOS		PCOS	
Outcomes obtained by the Smart Predictor for each Test Case	**Prediction: PCOS** Probability: 0.705882		**Prediction: PCOS** Probability: 0.647059		**Prediction: Not PCOS** Probability: 0.647059		**Prediction: Not PCOS** Probability: 0.529412		**Prediction: PCOS** Probability: 0.647059	

As illustrated in Table 5, the Smart Predictor correctly identified all five test cases, offering the corresponding probabilities and feature contributions for each input across all instances. The Smart Predictor's predictions were found to be consistent with the medical expert's review, classifying three cases as PCOS and two as not PCOS, with corresponding probabilities reflecting the confidence level of each prediction.

Furthermore, individual contributions of all 17 features selected by TOMIM to the prediction process, based on the given inputs, were outlined in the results of the smart predictor. Notably, Waist, Fast Food, and Skin darkening exhibited negative contributions, while other features, including follicle numbers (R), (L), and various parameters, contributed positively to the prediction.

Furthermore, the XAI-based smart predictor was deployed using the proposed two-level random forest classifier, incorporating features selected by the TOPCA (10 features), OSSM (15 features). Five different situations with input features were used to test the system, and each test produced predictions and matching contribution outcomes for each of the three feature selection techniques.

Table 6 shows that the smart predictor developed with TOMIM produced perfect predictions in every scenario. The OSSM-based smart predictor exhibited erroneous misclassifications in two instances and the TOPCA-based smart predictor misclassified one instance. Remarkably, the Random Forest Classifier model with two levels, employing feature sets derived from TOMIM, exhibited higher overall model accuracy in comparison to TOPCA and OSSM.

**Table 6:** Smart Predictor Predictions (correct prediction - fx1, incorrect prediction - fx2 and Prediction Probabilities.

Test Cases	Medical Expert Review	TOPCA method Pred.	TOPCA method Prob.	OSSM method Prob.	OSSM method Prob.	TOMIM method Prob.	TOMIM method Prob.
		10 features	10 features	15 features	15 features	17 features	17 features
1	PCOS		0.56		0.73		0.70
2	PCOS		0.64		0.53		0.64
3	Not PCOS		0.90		0.60		0.64
4	Not PCOS		0.51		0.66		0.52
5	PCOS		0.90		0.9		0.64

Furthermore, it outperformed in aligning with the predictions of the smart predictor as expressed in Table 6.This emphasizes the robustness and effectiveness of the smart predictor in accurate PCOS detection, especially when incorporating features selected by TOMIM.

## 4 Results and findings

In this research, using the numerical and clinical parameter dataset, the efficacy of four predictive models - two stacking ensemble models, a two-level random forest classifier ensemble, and classic (7 classifiers) for PCOS identification were investigated. Three techniques (TOPCA, OSSM, and TOMIM) were used, each of which chose 10, 15, and 17 features from the dataset's 40 features in order toemphasize optimal feature selection.

Based on the characteristics selected by each selection procedure, each classifier's performance was evaluated. To examine the effectiveness of the prediction analysis, the performance of each classifier using various feature sets was examined using five performance measures: specificity, F1 score, accuracy, precision, recall (sensitivity), and recall (sensitivity) [51].

The performance metrics examined the proportion of accurate and inaccurate predictions from the training sample, which was separated into four categories, and were mostly based on a comparison of expected and actual values: True Positive (TP): a situation in which the values expected and actual are both positive; False Positive (FP) is when the expected result is positive but the real value is negative; True Negative (TN) is when the original and anticipated values are both negative; and False Negative (FN) is when the actual value is positive but the predicted result is negative. The performance metrics in Equations (5), (6), (7), (8), (9) serve as a representation of these evaluations.

(5) $$Accuracy=\frac{TP+TN}{TP+TN+FP+FN}$$

(6) $$Precision=\frac{TP}{TP+FP}$$

(7) $$Recall=\frac{TP}{TP+FN}$$

(8) $$F1\;score=\frac{2\times Precision\times Recall\;}{Precision+Recall}$$

(9) $$Specificity=\frac{TN}{TN+FP}$$

In addition, the Matthew's correlation coefficient (MCC) was employed in the assessment of classifier performance [52]. When the binary predictor correctly predicts most positive and negative data occurrences, MCC is the only binary classification metric that produces a high score. It is in the range [-1.+1]; perfect misclassification is represented by extreme values of −1 and perfect classification is represented by extreme values of +1. For a coin-tossing classifier, an MCC value of 0 is anticipated. The MCC is calculated using formula 10:

(10) $$MCC=\frac{\left(TP\times TN\right)-\left(FP\times FN\right)}{\sqrt{\left(TP+FP\right)\left(TP+FN\right)\left(TN+FP\right)\left(TN+FN\right)}}$$

In this study, MCC was used only to evaluate the effectiveness of the three feature selection techniques on the two-level random forest classifier.

Table 7, Table 8, Table 9, Table 10 display the evaluation metrics, as defined in Equation (5) (10), for all classifiers using features selected through TOPCA, OSSM, and TOMIM methods. The top-performing results are that of the proposed 2 level random forest classifier using the TOMIM feature selection method, as emphasized in Table 9, Table 10.

Analyzing the evaluation results from Table 7, Table 8, Table 9, Table 10, it can be observed that the performances of the classifiers enhance significantly using the proposed two-level random forest ensemble method.

**Table 7:** Metrics of evaluation of different models using TOPCA method of feature selection (10 features).

MODEL	ACCURACY	PRECISION	RECALL	F1 SCORE	SPECIFICITY
Logistic Regression	76.1	70.9	79.5	75	73.3
Support Vector Machines	87.1	82.4	90.8	86.4	84.1
Decision Tree	80.6	79.4	79.4	79.4	81.8
Random Forest	86.2	84.6	84.6	84.6	87.5
Naive Bayes Classifier	69.7	62.1	83.6	71.3	58.3
K - Nearest Neighbour	86.2	79.2	95.5	86.6	77.9
AdaBoost	82.5	79.4	82.6	80.9	82.5
XGBoost	86.2	85.4	83.6	84.5	88.3
Extratrees	89.4	85.7	91.8	88.6	87.5
Stacking Ensemble 1	84.4	80.7	85.7	83.1	83.3
Stacking Ensemble 2	86.8	87.6	83.8	85.7	89.6
Proposed 2 Level Random Forest Classifier	**96.3**	96.8	94.8	95.8	97.5

**Table 8:** Metrics of evaluation of different models using OSSM method of feature selection (15 features).

MODEL	ACCURACY	PRECISION	RECALL	F1 SCORE	SPECIFICITY
Logistic Regression	86.8	82.6	91.1	86.7	83.1
Support Vector Machines	88.2	90.4	83.8	87.02	92.2
Decision Tree	83.4	82.3	82.3	82.3	84.4
Random Forest	87.6	83.1	90.8	86.8	85
Naive Bayes Classifier	63.3	55.2	96.6	70.3	35.8
K - Nearest Neighbour	91	86.6	95.5	90.9	87
AdaBoost	86.2	83.3	88.2	85.7	84.4
XGBoost	87.5	84.7	89.7	87.1	85.7
Extratrees	88.9	84.2	94.1	88.8	84.4
Stacking Ensemble 1	91	87.6	94.1	90.7	88.3
Stacking Ensemble 2	85.5	82.1	88.2	85.1	83.1
Proposed 2 Level Random Forest Classifier	**98.6**	100	96.9	98.4	100

**Table 9:** Metrics of evaluation of different models using TOMIM method of feature selection (17 features).

MODEL	ACCURACY	PRECISION	RECALL	F1 SCORE	SPECIFICITY
Logistic Regression	87.5	81.2	95.5	87.8	80.5
Support Vector Machines	92.6	87.9	96.9	92.2	89.1
Decision Tree	86.2	80.3	91.8	85.7	81.6
Random Forest	91.7	89.2	92.8	90.9	90.8
Naive Bayes Classifier	87.1	81.2	92.8	86.6	82.5
K - Nearest Neighbour	88	79.5	98.9	88.1	79.1
AdaBoost	89.9	88.7	88.7	88.7	90.8
XGBoost	93.5	92.8	92.8	92.8	94.1
Extratrees	93.1	90.2	95.5	92.8	90.9
Stacking Ensemble 1	91.7	90	91.8	90.9	91.6
Stacking Ensemble 2	93.1	91	93.8	92.4	92.5
Proposed 2 Level Random Forest Classifier	**99.31**	100	98.5	99.2	100

**Table 10:** Matthew's correlation coefficient of different models using the feature selection methods.

MODEL	TOPCA	OSSM	TOMIM
Proposed 2 Level Random Forest Classifier	92.5	97.2	**98.5**

**Fig. 15:**
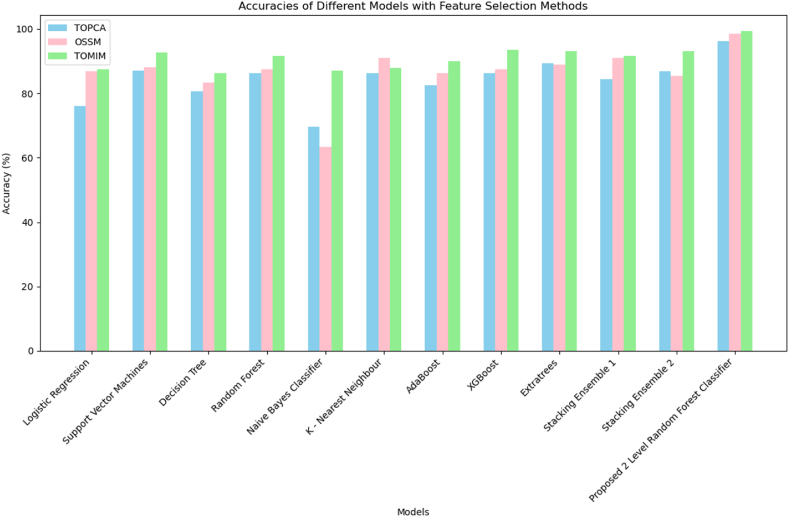
Comparative accuracy analysis of the models using TOPCA, OSSM and TOMIM methods.

Fig. 15 illustrates that the proposed 2-level random forest ensemble achieves an accuracy of **99.31 %** with 17 features selected by the Threshold-driven Optimized Mutual Information Method. The classifier also demonstrated an accuracy of **98.6%**with 15 features selected by the Optimized Salp Swarm method, which is reasonably competitive with the threshold-driven optimized mutual information method. Using the 10 features selected by the Threshold-driven Optimized Principal Component Analysis method, the 2-level random forest ensemble achieved **96.3 %** accuracy. The Explainable Artificial Intelligence-based PCOS classifier was thereby implemented using the two level random forest classifier with features selected from the Threshold-driven Optimized Mutual Information Method.

### 4.1 Results of 8 - fold cross-validation on the proposed two level random forest classifier

The PCOS classification routine utilizing the proposed two-level random forest classifier was iterated 25 times across 8 folds of the dataset, and the resulting overall average accuracy was evaluated for statistical significance. A one-sample *t*-test was conducted under the assumption of no disparity in mean accuracy scores among the 25 runs, establishing a hypothetical mean equivalent to the initial accuracy obtained.

Table 11 displays the accuracy for each individual run and the overall average accuracy for all the 25 runs. As in Table 12, the T-statistic measured the difference between the overall average accuracy of the model across 25 runs and the actual model accuracy to be 0.39. This value, along with the associated P-value of 0.70, indicates that the observed difference is not statistically significant. Since the p-value exceeds 0.05, there's insufficient evidence to conclude that the mean accuracy derived from the 25 runs significantly differsfromthe expected values.

**Table 11:** Classification results of 25-run 8-fold cross validation when training and testing the two level random forest classifier.

Run	Mean Accuracy (%)
1	99.31
2	99.3
3	99.58
4	99.31
5	99.31
6	99.3
7	99.44
8	99.31
9	99.72
10	98.89
11	99.16
12	99.17
13	1.0
14	99.31
15	99.31
16	99.16
17	99.3
18	99.44
19	99.31
20	99.03
21	99.17
22	98.61
23	99.58
24	99.58
25	99.3
Overall	99.32

**Table 12:** Statistical results of 25-run 8-fold cross validation when training and testing the two level random forest classifier.

Factor	Result for Accuracy
Mean	99.32
Variance	0.0000706
Observations	25
T-statistic	0.3884
p-value	0.701
Confidence interval at 95 % level of confidence	(0.9922, 0.9943)

Additionally, to assess the precision of the mean accuracy estimate, a confidence interval at the several confidence levels were computed and the confidence interval at most commonly used 95 % level of confidence us presented in Table 12.

The comprehensive evaluation of the model's performance through 8-fold cross-validation over 25 runs yielded a mean accuracy of approximately 0.9932. The variance of the accuracy scores was relatively low, indicating consistent performance across different runs.

Notably, the 95 % confidence interval for the mean accuracy ranged from 0.9922 to 0.9943. This narrow interval suggested a high degree of precision in the accuracy estimate, reinforcing the robustness of the model.

The *t*-test results, with a t-statistic of 0.388 and a p-value of 0.701, indicated no significant difference between the sample mean and the hypothetical mean of 0.993, further underscoring the reliability of the model's performance. The consistent accuracy, combined with a narrow confidence interval, indicates that the model is both highly effective and reliably replicable. This demonstrates its strong predictive capabilities for identifying the target variable with minimal performance variability, further emphasizing the statistical significance of the cross-validation results.

### 4.2 Impact and clinical relevance of feature selection

The Kaggle PCOS dataset employed in this research comprised 44 features, each essential for the precise diagnosis of PCOS, as corroborated by the literature. The significance of key features that were selected by the proposed Threshold-driven Optimal Mutual Information Method (TOMIM) in clinical settings are as follows:

The follicle count in the left and right ovaries, denoted as **Follicle No. (L) and Follicle No. (R),** serves as a key indicator of polycystic ovarian morphology, a defining feature of PCOS [53]. An increased count, generally 12 or more follicles per ovary according to the Rotterdam criteria, is a primary diagnostic marker. In test case 1 (Table 5), the PCOS smart predictor assigned positive contribution scores of 0.010553 for 10 follicles in the right ovary and 0.015817 for 7 in the left, underscoring the strong link between follicle count and PCOS. This emphasizes the role of follicle count in both model predictions and clinical assessments, where a high count typically prompts further investigation and intervention.

Average follicle sizes, **Avg. F size (L)** and **Avg. F size (R)**) are critical for understanding ovarian morphology and improving PCOS diagnosis accuracy [54]. Follicle sizes exceeding 9 mm often indicate multiple immature follicles, a common feature in PCOS. In test case 1 (Table 5), the model assigns contribution scores of 0.031168 for an average follicle size of 6 mm in the left ovary and 0.015676 for 9 mm in the right, highlighting the significance of these measurements. These scores demonstrate how average follicle size influences both clinical assessments and the model's predictions, reinforcing its role in diagnosing PCOS.

**Cycle length (days)** and **Cycle regularity(R/I)** are essential markers of ovulatory dysfunction in PCOS, often indicating hormonal imbalances [55]. Prolonged or irregular cycles, typically lasting more than 35 days, are frequently linked to PCOS symptoms such as hyperandrogenism and the presence of polycystic ovaries. In test case 1 (Table 5), the PCOS Smart predictor assigns contribution scores of 0.071869 for a 5-day cycle length and 0.002526 for cycle irregularity, highlighting their significance in diagnosis. These menstrual irregularities are critical in the model's prediction of a higher probability of PCOS, demonstrating their key role in both clinical evaluation and model outcomes.

Anthropometric measurements like **Waist (inch)**, **Hip (inch)** circumference, **Waist-Hip Ratio**, and **Body Weight (Kg)** play a key role in evaluating central obesity, a common feature of PCOS often associated with metabolic issues such as insulin resistance [56]. Central obesity heightens the risk of metabolic syndrome, guiding treatment strategies to manage insulin resistance and lower cardiovascular risk. In test case 1 (Table 5), the model assigns a positive contribution score of 0.091081 for a hip size of 35, indicating central adiposity linked to hormonal imbalances in PCOS. Meanwhile, a waist size of 30 with a negative contribution of −0.65842 points to an elevated risk of insulin resistance and metabolic dysfunction. A waist-hip ratio of 0.9, contributing 0.001439, reflects fat distribution relevant to PCOS, and a weight of 60 kg, contributing 0.012733, adds a moderate impact on the prediction. Collectively, these measurements underscore the crucial role of central obesity in diagnosing and predicting PCOS.

While not directly associated with PCOS, hemoglobin levels, **Hb (g/dl)** offer valuable insights into overall health and potential blood disorders that could affect PCOS management [57]. For example, anemia, indicated by low hemoglobin levels, may require treatment as it can exacerbate fatigue and other common PCOS symptoms. In test case 1 (Table 5), the PCOS smart predictor assigned a positive contribution score of 0.020021 to hemoglobin levels, emphasizing its relevance in evaluating general health and metabolic conditions within the model's predictions.

**Age (yrs)** is a key factor in determining the symptoms and treatment strategies for PCOS. Younger women are more likely to experience significant menstrual irregularities, while older women may develop more severe metabolic issues such as insulin resistance and dyslipidemia [58]. Tailoring treatments to address these age-specific challenges is essential for effective management of PCOS. In test case 1 (Table 5), the model assigns a positive score of 0.035211 to an age of 25, showing how age impacts the likelihood of a PCOS diagnosis. This underscores the model's ability to incorporate age into its predictions, helping to guide personalized treatment plans.

Lifestyle factors such as **Fast Food (Y/N)** intake and recent **Weight Gain (Y/N)** exacerbate PCOS symptoms by increasing insulin resistance and disrupting hormonal balance. Advising patients on dietary modifications and healthier lifestyle choices is essential for effective PCOS management [59]. In test case 1 (Table 5), the model attributes a contribution score of 0.054673 to weight gain, underscoring its considerable effect on obesity-related factors, while fast food consumption has a score of −0.002149, indicating its influence on dietary practices. A patient who has recently gained weight is more likely to be diagnosed with PCOS, while frequent fast food consumption might slightly decrease this likelihood, illustrating how these lifestyle factors impact the model's predictions.

Clinical signs such as **Skin darkening (Y/N)**, especially Acanthosis nigricans, are indicative of insulin resistance, commonly associated with PCOS. Identifying these signs is crucial for shaping treatment approaches that improve insulin sensitivity and reduce metabolic risks [60]. In test case 1 (Table 5), the absence of skin darkening correlates with a contribution score of −0.156572, indicating a lower risk of PCOS diagnosis linked to reduced insulin resistance. Thus, when a patient does not show skin darkening, the model revises its prediction to suggest a decreased probability of PCOS, underscoring the important role of this clinical sign in diagnostic assessments.

Although **Blood Group** is not directly associated with PCOS, incorporating it into patient profiles improves comprehensive health evaluations. Exploring potential links between blood groups and the occurrence or severity of PCOS can facilitate the creation of personalized treatment plans and a more holistic approach to patient care [61]. In test case 1 (Table 5), the blood group positively influences the model's predictions, with a contribution score of 0.043892, although the impact is minimal.

**Marriage status** and duration provide important insights for fertility planning and reproductive health in managing PCOS. For married individuals, particularly those aiming to conceive, evaluating PCOS symptoms and exploring fertility treatment options are crucial [62]. Customizing counseling and treatment strategies based on marital status and fertility goals can enhance reproductive outcomes. In test case 1 (Table 5), the PCOS smart predictor assigns a positive contribution score of 0.050762 to a marriage duration of 2 years, indicating its relevance to fertility planning and necessary medical consultations. For example, a married patient experiencing difficulties in conceiving might receive an elevated score for this factor, highlighting the significance of targeted medical guidance and intervention in the model's predictions.

The combined contributions of various features led to a PCOS-positive prediction for test case 1, yielding a probability of 70.58 %, a trend consistently reflected in other test cases. This underscores the efficacy of the proposed PCOS smart predictor in utilizing essential features for an in-depth assessment of PCOS, facilitating accurate diagnosis and effective management. The outcomes of the model emphasize the therapeutic significance of each attribute. Additionally, the features selected by TOMIM have been rigorously validated through thorough literature reviews, expert consultations, and careful evaluation of model performance, reinforcing their strong relevance in PCOS prediction.

### 4.3 Performance of the smart predictor on other comparable feature-driven clinical datasets

The performance of the Smart Predictor was evaluated using four distinct clinical datasets aimed at predicting breast cancer, chronic kidney disease, heart disease, and lung cancer, all derived from similar feature-driven datasets. These real-time, open-source datasets were sourced from the Kaggle data repository. Each dataset utilized clinical feature values to predict the presence or absence of the disease, following the same methodology applied to the PCOS dataset in this research.

#### 4.3.1 Prediction of breast cancer using the proposed smart predictor and breast cancer Wisconsin (diagnostic) dataset

The Breast Cancer Wisconsin (Diagnostic) Dataset is a key resource on Kaggle for predicting the presence of breast cancer [63], maintained by the UCI Machine Learning Repository. Initially provided by Dr. William H. Wolberg from the University of Wisconsin Hospitals, this widely recognized dataset is freely available through the UCI Machine Learning Repository and is extensively used in educational and research applications [64].

It consists of 569 samples from fine needle aspirate (FNA) tests of breast masses, each containing 30 numerical attributes derived from digitized images of the masses, with 357 benign and 212 malignant cases. These attributes describe the cell nuclei and are categorized into three types: mean, standard error, and the worst (largest) value for each attribute. The dataset includes labels indicating whether the diagnosis is benign or malignant, which serves as the target variable for classification tasks. It is commonly used in machine learning to develop models for accurate breast cancer diagnosis.

Table 13, Table 14 highlight the results achieved by the proposed Smart predictor in breast cancer prediction using this dataset.

**Table 13:** Results of feature selection and classification on theBreast Cancer Wisconsin (Diagnostic) Dataset using the proposed methodology.

Number of features selected by TOMIM feature selection method	9
Accuracy of the feature selection method on the validation set	93.7 %
Features selected by the TOMIM feature selection method	**radius_mean:** Average distance from the center to the nucleus boundary.
	**perimeter_mean:** Average length of the nucleus boundary.
	**area_mean:** Average area of the nucleus.
	**concave points_mean:** Average number of indentations in the nucleus boundary
	**area_se:** Variation in the area measurement.
	**radius_worst:** Largest radius measurement.
	**perimeter_worst:** Largest perimeter measurement.
	**area_worst:** Largest area measurement.
	**concave points_worst:**Most number of indentations in the nucleus boundary.
Accuracy of the classifier	97.90 %
Overall K – Fold Cross Validation accuracy of the classifier (8 folds, 25 runs)	98.17 %

**Table 14:** Smart predictor's predictions on unseen data for breast cancer diagnosis.

Selected Features	Test Case 1		Test Case 2	
	Value	Contribution	Value	Contribution
radius_mean	7.43	−0.005994	17.02	0.016018
perimeter_mean	60.33	0.017041	129.4	0.038261
area_mean	271.8	0.011369	850.00	0.082043
concave points_mean	0.0205	−0.242885	0.1875	−0.071472
area_se	15.5	0.125472	112.5	0.095892
radius_worst	9.34	0.044436	19.46	0.037211
perimeter_worst	64.34	−0.002486	117.8	−0.009782
area_worst	304.8	0.09326	1745.08	0.21393
concave points_worst	0.0534	−0.090668	0.1556	−0.091239
Expected Class	Benign		Malignant	
Predicted class and probability obtained by the Smart Predictor for each Test Case	**Prediction: Benign** Probability: 0.555556		**Prediction: Malignant** Probability: 0.888889	

#### 4.3.2 Prediction of chronic kidney disease using the proposed smart predictor and chronic kidney disease (CKD) dataset

Originating from a hospital in Karaikudi, Tamil Nadu, India, the Chronic Kidney Disease (CKD) dataset comprises 400 records with 25 attributes, making it ideal for classification tasks [65]. This dataset, initially available through the UCI Machine Learning Repository and commonly referred to as the CKD dataset, features 24 attributes plus one target variable, categorized into two classes: yes or no.

Hosted on Kaggle, this dataset is a valuable tool for predicting chronic kidney disease. It includes both numerical and categorical data related to a range of patient health indicators, such as blood pressure, blood glucose levels, and red blood cell count. The target variable indicates the presence or absence of chronic kidney disease.

This dataset is widely used in machine learning projects to develop models that aid in the early detection and diagnosis of the condition [66] and was preprocessed to handle missing values and correct inaccuracies before being fed into the Smart Predictor.

Table 15, Table 16 highlight the results achieved by the proposed Smart predictor in breast cancer prediction using this dataset.new.

**Table 15:** Results of feature selection and classification on the Chronic Kidney Disease Dataset using the proposed methodology.

Number of features selected by TOMIM feature selection method	8
Accuracy of the feature selection method on the validation set	98.75 %
Features selected by the TOMIM feature selection method	**Specific Gravity**: Reflects the kidney's capacity to concentrate urine.
	**Albumin:** Presence of protein in urine, a sign of kidney damage.
	**Serum Creatinine**: Byproduct in blood, elevated levels indicate kidney dysfunction.
	**Hemoglobin**: Protein that carries oxygen, typically reduced in kidney disease.
	**Packed Cell Volume**: Proportion of red blood cells, decreased in anemia.
	**Red Blood Cell Count**: Quantity of red blood cells, lowered in anemia.
	**Hypertension**: Elevated blood pressure, commonly associated with kidney disease.
	**Diabetes Mellitus**: Blood sugar disorder, a primary cause of kidney disease.
Accuracy of the classifier	97 %
Overall K – Fold Cross Validation accuracy of the classifier (8 folds, 25 runs)	99.36 %

**Table 16:** Smart predictor's predictions on unseen data for Chronic Kidney Disease prediction.

Selected Features	Test Case 1		Test Case 2	
	Value	Contribution	Value	Contribution
Specific Gravity	1.013	0.079973	1.01	−0.05625
Albumin	0.0167	0.017041	0	0.0675
Serum Creatinine	1	−0.00672	0.5	0.18375
Hemoglobin	6.6	0.018756	13	0.045673
Packed Cell Volume	43	0.060073	40	−0.07
Red Blood Cell Count	0.2671	0.075754	6.2	−0.07
Hypertension	1	0.081243	0	0.07
Diabetes Mellitus	0	−0.159274	0	0.043764
Expected Class	Disease		Not Disease	
Predicted class and probability obtained by the Smart Predictor for each Test Case	**Prediction:Disease** Probability: 0.75		**Prediction:Not Disease** Probability: 0.625	

#### 4.3.3 Prediction of heart disease using the proposed smart predictor and heart disease dataset

The Heart Disease dataset, integrates data from Cleveland, Hungary, Switzerland, and Long Beach V. While it originally contains 76 attributes, research primarily emphasizes 14 crucial features [67,68]. The "target" variable reflects the presence of heart disease (0 = no disease, 1 = disease). This dataset encompasses a range of patient details and diagnostic measures, such as age, sex, chest pain type, cholesterol levels, among others.

Table 17, Table 18 highlight the results achieved by the proposed Smart predictor in heart disease prediction using this dataset.

**Table 17:** Results of feature selection and classification on the Heart Disease Dataset using the proposed methodology.

Number of features selected by TOMIM feature selection method	7
Accuracy of the feature selection method on the validation set	98.75 %
Features selected by the TOMIM feature selection method	**cp (Chest Pain Type):** Represents the category of chest pain experienced, divided into four types.
	**chol (Cholesterol):** Indicates the serum cholesterol level in mg/dl, a significant risk factor for heart disease.
	**thalach (Maximum Heart Rate Achieved):** The peak heart rate reached during exercise, reflecting cardiovascular fitness.
	**exang (Exercise Induced Angina):** Identifies whether chest pain occurs during physical exertion (1 = yes, 0 = no).
	**oldpeak (ST Depression):** Measures ST segment depression caused by exercise, compared to rest, signaling potential heart stress.
	**ca (Number of Major Vessels):** Counts the major blood vessels (0–3) visible by fluoroscopy, indicating blood flow levels.
	**thal (Thalassemia):** A blood disorder categorized here to show normal, fixed, or reversible defects.
Accuracy of the classifier	98.65 %
Overall K – Fold Cross Validation accuracy of the classifier (8 folds, 25 runs)	99.89 %

**Table 18:** Smart predictor's predictions on unseen data for Heart Disease prediction.

Selected Features	Test Case 1		Test Case 2	
	Value	Contribution	Value	Contribution
cp	0	−0.00692	0	−0.00692
chol	248	0.2547	124	0.300197
thalach	122	0.165906	167	0.16351
exang	0	−0.096396	0	−0.098791
oldpeak	1	0.091009	1	0.091009
ca	0	−0.095334	2	0.006817
thal	2	0.005465	3	0.005465
Expected Class	Disease		Not Disease	
Predicted class and probability obtained by the Smart Predictor for each Test Case	**Prediction:Disease** Probability: 0.714286		**Prediction:Not Disease** Probability: 0.857143	

#### 4.3.4 Lung cancer detection using the proposed smart predictor and heart disease dataset

The Lung Cancer dataset available on Kaggle contains 16 features and 309 records [69], integrating both numerical and categorical variables that capture detailed patient information and diagnostic insights [70]. This comprehensive dataset, which includes aspects like age, smoking behavior, and medical history, is valuable for building predictive models for lung cancer. The presence of categorical variables enhances the understanding of patient profiles, contributing to the analysis of disease patterns and associated risk factors.

Table 19, Table 20 highlight the results achieved by the proposed Smart predictor in heart disease prediction using this dataset.

**Table 19:** Results of feature selection and classification on the Lung Cancer Detection Dataset using the proposed methodology.

Number of features selected by TOMIM feature selection method	5
Accuracy of the feature selection method on the validation set	87.96 %
Features selected by the TOMIM feature selection method	**AGE:** Represents the patient's age, an important factor in evaluating the risk of lung cancer.
	**ALLERGY:** Shows if the patient has any allergies, which might be relevant in assessing respiratory health**.**
	**WHEEZING:** Indicates whether the patient experiences wheezing, a symptom commonly associated with respiratory conditions.
	**ALCOHOL CONSUMING:** Reflects the patient's alcohol consumption habits, which can impact the risk of developing lung cancer.
	**COUGHING:** Denotes whether the patient has a persistent cough, a symptom often related to lung cancer and other respiratory illnesses.
Accuracy of the classifier	94.44 %
Overall K – Fold Cross Validation accuracy of the classifier (8 folds, 25 runs)	91.48 %

**Table 20:** Smart predictor's predictions on unseen data for Lung Cancer Detection.

Selected Features	Test Case 1		Test Case 2	
	Value	Contribution	Value	Contribution
AGE	70	0.152596	21	−0.138947
ALLERGY	1	0.02058	0	0.067836
WHEEZING	0	−0.084328	1	0.032749
ALCOHOL CONSUMING	1	0.270334	0	0.027193
COUGHING	1	0.031863	0	0.067836
Expected Class	Disease		Not Disease	
Predicted class and probability obtained by the Smart Predictor for each Test Case	**Prediction:Disease** Probability: 0.8		**Prediction:Not Disease** Probability: 0.87	

The evaluation results highlight the efficacy of the proposed methodology in precisely predicting a range of diseases using similar feature-based frameworks. This confirms that the Smart Predictor is both robust and dependable, making it highly applicable for disease prediction in diverse clinical environments through feature-driven approaches.

## 5 Discussion

In order to distinguish between PCOS and non-PCOS data, this research examines three machine learning ensemble methodologies that combine several classifiers with conventional machine learning approaches.

### 5.1 Comparison with state-of-the-art classification techniques in PCOS domain

Traditional machine learning classifiers were the main focus of previous studies, but more recently, researchers have begun investigatingensemble strategies for PCOS detection, using common ensemble models such as bagging, boosting or voting.

### 5.2 Advantages and expansion over existing PCOS prediction studies

Within the realm of PCOS prediction, this study introduces a pioneering approach through the development of an innovative smart predictor using the Shapash library. By employing a two-level random forest ensemble method combined with eXplainable Artificial Intelligence (XAI), the proposed model achieves unparalleled transparency and explainability.

This research significantly contributes to the field by providing in-depth discussions on the clinical significance of each feature, thereby enhancing the interpretability of the prediction model.Compared to traditional classifiers and recent stacking ensemble methods referenced in the literature ([[71], [72], [73], [74], [75], [76], [77], [78], [79], [80], [81]]), the proposed two-level Random Forest classifier demonstrates exceptional performance.

As shown in Table 21 of Section 5.1, it consistently achieves an accuracy of 99.31 % across an 80:20 train-test split and a cross-validation accuracy score of 99.32 % over 25 runs on the Kaggle PCOS dataset.

**Table 21:** Comparative analysis of performance of various state-of-the-art techniques for PCOS prediction using the same Kaggle PCOS dataset.

References	Classification Technique	Data Split Ratio (Train:Test)	Accuracy
Manjal et al. (2020) [71]	Extratree	70:30	88 %
Denny et al. (2019) [72]	Random Forest	80:20	89.02 %
Bharati et al. (2020) [73]	Hybrid Random Forest and Logistic Regression	60:40	91.01 %
P.Chitra et al. (2022) [74]	Artificial Neural Network	80:20	92 %
Panda et al. (2024) [75]	Random Forest	80:20	92 %
Hdaib et al. (2022) [76]	Linear Discriminant Classifier	80:20	92.60 %
Tiwari et al. (2022) [77]	Random Forest	70:30	93.25 %
Rahman et al. (2024) [78]	Random Forest and AdaBoost	80:20	94 %
Sayma Alam Suha et al. (2023) [79]	Stacking Ensemble	70:30	95.7 %
Inan et al. (2021) [80]	Extreme Gradient Boosting	80:20	95.83 %
Khanna et al. (2023) [81]	Stacking Ensemble	80:20	98 %
Proposed Method	Two Level Random Forest Classifier	80:20	**99.31 %**

Additionally, the method accurately classified five test cases meticulously simulated in consultation with medical experts, ensuring alignment with real-time data. This superior performance is attributed to the advanced ensemble learning approach and meticulous optimization process, which incorporates Mutual Information-based feature selection and a novel threshold-based Principal Component Analysis (PCA).

Notably, the Threshold-driven Optimized Mutual Information Method (TOMIM) identified an optimal set of 17 features, significantly enhancing classifier performance compared to alternative selection methods. These 17 features, classified as real-time clinical parameters, are crucial for expert clinicians in predicting PCOS, highlighting the model's clinical relevance and practical applicability in healthcare contexts.

The PCOS prediction literature typically lacks the strong incorporation of such an explainable technique and discussions of feature relevance in clinical settings, a gap that this study addresses comprehensively. This methodology is also applicable to various other feature-driven datasets, demonstrating its versatility and potential for broader applications.

### 5.3 Comparison with state-of-the-art classification techniques in other clinical domains

Similar machine learning models have been applied across various healthcare and disease prediction domains. Rahman et al. [82] developed a heart disease prediction method using multiple machine learning algorithms on a dataset of 1024 patients, achieving 99 % accuracy with decision tree and random forest classifiers. Nagavelli et al. [83] created a clinical decision support system for heart disease prediction utilizing an Extreme Gradient Boosting classifier, which achieved 95.9 % accuracy.

Das et al. [84] introduced the Machine Learning-Based Intelligent System for Breast Cancer Prediction, employing Boruta for feature selection and their MLISBCP classification model, resulting in a 97.53 % accuracy for early breast cancer detection. Islam et al. [85] used an Extreme Gradient Boosting classifier to predict chronic kidney disease, achieving 98.3 % accuracy on a dataset with 24 features and 400 instances.

Ibrahim et al. [86] proposed a late fusion-based machine learning model for predicting β-Thalassemia carriers, achieving 96 % accuracy on a dataset with 5066 instances. Alatrany et al. [87] attained 98.9 % accuracy in diagnosing early Alzheimer's disease using a dataset of 169,408 records and 1024 features, enhancing explainability with rule-extraction methods validated by SHAP and LIME models.

DeGroat et al. [88] employed an innovative soft voting ensemble classifier, incorporating four ML classifiers, to analyze the complete transcriptome of CVD patients, achieving up to 96 % accuracy and identifying 18 key biomarkers as early indicators of CVD. Hassoon et al. [89] utilized a tongue color dataset comprising 5260 images categorized into seven distinct color classes and tested these using six different machine learning algorithms. XGBoost emerged with the highest accuracy at 98.71 %, making it the classifier of choice for real-time prediction of tongue color and associated disease diagnosis.

Laghmati et al. [90] developed a CAD system for breast cancer classification using PCA for feature selection, grid search for hyperparameter tuning, and cross-validation. They employed seven ML classifiers and ensemble models with Majority Voting and Stacking with Logistic Regression (S-LR). The system achieved over 96 % recall with XGBoost for the Mammographic Mass dataset and 97.37 % accuracy with S-LR for the Wisconsin Breast Cancer Dataset. Reza et al. [91] proposed two stacking-based models for diabetes classification utilizing the PIMA Indian diabetes dataset, simulated data, and additional data from a local healthcare facility. They employed both classical and deep neural network stacking ensemble methods, validated through train-test and cross-validation (CV) techniques. The models achieved an impressive 95.50 % accuracyscore using 5-fold CV on the simulation study.

### 5.4 Strength and scope compared to other disease prediction studies

From the above discussion in Section 5.3, it is visible that recent advancements in various clinical domains have embraced state-of-the-art machine learning techniques for classifying medical data, including numerical features and image datasets, across diverse disease predictions. Traditional machine learning classifiers and ensembles have played crucial roles in these breakthroughs.

Alternatively, this study introduces a novel two-level random forest classification ensemble for PCOS prediction, marking a pioneering approach not explored in previous research, achieving an outstanding accuracy of 99.31 % and cross-validation accuracy of 99.32 %. Beyond superior accuracy, this approach innovatively integrates model explainability, unraveling the nuanced contributions of each feature to predictive outcomes.

By meticulously examining the clinical implications of selected features, this research enhances transparency and underscores practical applications for feature-driven PCOS diagnosis in clinical settings. This methodology holds promise for advancing diagnostic outcomes across a spectrum of medical conditions reliant on feature-driven analysis.

## 6 Conclusion

The innovative smart predictor presented in this study has the potential to transform PCOS detection by harnessing machine learning and explainable AI techniques. By integrating this tool into clinical practice, healthcare providers can achieve more precise and efficient diagnoses based on patient symptoms and test results. Early detection enabled by the smart predictor could enhance reproductive health outcomes for women through tailored treatment plans. Utilizing eXplainable AI and a robust two-level random forest ensemble model, with features selected through the Threshold-driven Optimized Mutual Information Model, this tool alleviates the workload on physicians and enables quicker decision-making. Its application, particularly in rural healthcare settings, could bridge the gap in specialized care, making PCOS diagnosis more accessible.

Furthermore, there were certain limitations encountered during the study. When constructing the Smart Predictor using two stacking ensemble approaches, issues related to compatibility and potential biases in model integration were observed. The limited dataset size and scope may have also contributed to biases, reducing the generalizability of the results. To improve robustness, it is crucial to explore alternative ensemble methods like hybrid and dynamic ensembles.

Future research should incorporate a wider range of hormonal, clinical, metabolic, and genetic markers, including DENND1A variants and FSHR polymorphisms, as well as lifestyle data and advanced medical imaging, to enhance predictive accuracy and clinical relevance. Expanding the dataset to include longitudinal data would offer deeper insights into PCOS progression and treatment efficacy, helping to mitigate biases and provide a more comprehensive understanding of patient demographics and disease evolution. This approach can be applied to other women's health conditions as well. Improving the model's explainability with advanced techniques and visualizations will build trust among clinicians and patients. Additionally, refining the model to offer personalized treatment recommendations based on PCOS subtypes will support more targeted and effective therapeutic strategies.

## CRediT authorship contribution statement

**Umaa Mahesswari G:** Writing – review & editing, Writing – original draft, Methodology, Investigation, Formal analysis, Conceptualization. **Uma Maheswari P:** Supervision, Project administration.

## Data Availability

Data incorporated in the article are publicly available in the Kaggle repository and have been referenced as [35,64,65,67,69] in the article.

## Ethics declaration Statement

Review and/or approval by an ethics committee was not needed for this study because a publicly available open-source dataset was utilized for the experiment, and based on this dataset, the data for the five test cases discussed in Section 3.4.3 were exclusively in consultation with medical experts. This study did not involve direct participation of humans or animals.

## Funding Statement

The authors would like to thank the Centre for Research, Anna University for providing financial assistance through Anna Research Fellowship scheme.

## Declaration of competing interest

The authors declare that they have no known competing financial interests or personal relationships that could have appeared to influence the work reported in this paper.

## References

[1] Muslim Md, Zakwan Mohd (2024). Correlation between anti-mullerian hormone with insulin resistance in polycystic ovarian syndrome: a systematic review and meta-analysis. J. Ovarian Res..

[2] Stener-Victorin Elisabet (2024). Polycystic ovary syndrome. Nat. Rev. Dis. Prim..

[3] Salari Nader (2024). Global prevalence of polycystic ovary syndrome in women worldwide: a comprehensive systematic review and meta-analysis. Arch. Gynecol. Obstet..

[4] Suma K.G. (2022).

[5] Hajam Younis Ahmad (2024). A review on critical appraisal and pathogenesis of polycystic ovarian syndrome. Endocrine and Metabolic Science.

[6] McKenney Kathryn M. (2024). Severe maternal morbidity in polycystic ovary syndrome. Am. J. Obstet. Gynecol..

[7] Sikarwar Neetu Singh, Kazim Farhat (2024). Assessing the prevalence and implications of PCOS in women: a comprehensive study. Eur. J. Cardiovasc. Med..

[8] Das Ayushi, Choudhury Deepjyoti, Sen Arpita (2024). A collaborative empirical analysis on machine learning based disease prediction in health care system. Int. J. Inf. Technol..

[9] Dason Ebernella Shirin (2024). Diagnosis and management of polycystic ovarian syndrome. CMAJ (Can. Med. Assoc. J.).

[10] Shiwlani A., Khan M., Sherani A.M.K., Qayyum M.U., Hussain H.K. (2024). Revolutionizing healthcare: the impact of artificial intelligence on patient care, diagnosis, and treatment. JURIHUM: JurnalInovasi dan Humaniora.

[11] Sadeghi Z., Alizadehsani R., Cifci M.A., Kausar S., Rehman R., Mahanta P., Pardalos P.M. (2024). A review of explainable artificial intelligence in healthcare. Comput. Electr. Eng..

[12] Windisch P., Weber P., Fürweger C., Ehret F., Kufeld M., Zwahlen D., Muacevic A. (2020 Nov). Implementation of model explainability for a basic brain tumor detection using convolutional neural networks on MRI slices. Neuroradiology.

[13] Apostolopoulos Ioannis D., Apostolopoulos Dimitris J., Papathanasiou Nikolaos D. (2022). Deep learning methods to reveal important X-ray features in COVID-19 detection: investigation of explainability and feature reproducibility. Reports.

[14] Militello C., Prinzi F., Sollami G., Rundo L., La Grutta L., Vitabile S. (2023). CT radiomic features and clinical biomarkers for predicting coronary artery disease. Cognitive Computation.

[15] Mehr, Homay Danaei, Polat Huseyin (2022). Diagnosis of polycystic ovary syndrome through different machine learning and feature selection techniques. Health Technol..

[16] Zhang Xinyi (2021). Raman spectroscopy of follicular fluid and plasma with machinelearning algorithms for polycystic ovary syndrome screening. Mol. Cell. Endocrinol..

[17] Elmannai Hela (2023). Polycystic ovary syndrome detection machine learning model based on optimized feature selection and explainable artificial intelligence. Diagnostics.

[18] Medeiros de, Freitas Sebastião (2024). Anthropometric, metabolic, and endocrine parameters as predictors of estimated average glucose and other biomarkers of dysglycemia in women with different phenotypes of polycystic ovary syndrome. Horm. Metab. Res..

[19] Lavanya S., Ramya K., Vijayakumar R. (2024). Correlation between insulin resistance indices and endometrial thickness to predict metabolic syndrome & ovulatory dysfunction in phenotypes of polycystic ovarian syndrome in south Indian population. Educational Administration: Theory and Practice.

[20] Büyükyılmaz Gönül (2024). The role of the AMH, SHBG, free androgen index and LH/FSH ratio in the diagnosis of polycystic ovary syndrome in adolescent. Turkish Journal of Pediatric Disease.

[21] Pratama Gita (2024). Mechanism of elevated LH/FSH ratio in lean PCOS revisited: a path analysis. Sci. Rep..

[22] Łagowska Karolina, Bajerska Joanna, Maria Pieczyńska-Zając Joanna (2024). Dietary factors and the risk of depression among women with polycystic ovary syndrome. Nutrients.

[23] Shi Dongmei (2024). The effect of subclinical hypothyroidism on hormonal and metabolic profiles and ovarian morphology in patients with polycystic ovary syndrome: a cross-sectional study. Gynecol. Endocrinol..

[24] George S., Alex A. (2021). Assessment of symptoms and diet intake in young adult with polycystic ovary syndrome (PCOS). J. Sci. Res..

[25] Aggarwal S., Pandey K., Senior Member I. (2022). Determining the representative features of polycystic ovary syndrome via design of experiments. Multimed. Tools Appl..

[26] Patil Smital D., Deore Pramod J., Patil Vaishali Bhagwat (2024). An intelligent computer aided diagnosis system for classification of ovarian masses using machine learning approach. International Research Journal of Multidisciplinary Technovation.

[27] chitoTchapga, Mih T.A., TchagnaKouanou A., FozinFonzin T., Kuetche Fogang P., Mezatio B.A., Tchiotsop D. (2021). Biomedical image classification in a big data architecture using machine learning algorithms. J. Healthc. Eng..

[28] Teo Zhen Ling (2024). Federated machine learning in healthcare: a systematic review on clinical applications and technical architecture. Cell Reports Medicine.

[29] Prapty S., Shitu T.T. (2020). 2020 23rd International Conference on Computer and Information Technology (ICCIT), DHAKA, Bangladesh.

[30] Kodipalli A., Devi S. (2021 Nov 30). Prediction of PCOS and mental health using Fuzzy inference and SVM. Front. Public Health.

[31] Nasim S., Almutairi M.S., Munir K., Raza A., Younas F. (2022). A novel approach for polycystic ovary syndrome prediction using machine learning in bioinformatics. IEEE Access.

[32] Hdaib D., Almajali N., Alquran H., Mustafa W.A., Al-Azzawi W., Alkhayyat A. (2022). 2022 5th International Conference on Engineering Technology and its Applications (IICETA).

[33] Abu Adla Y.A., Raydan D.G., Charaf M.-Z.J., Saad R.A., Nasreddine J., Diab M.O. (2021). 2021 Sixth International Conference on Advances in Biomedical Engineering (ICABME).

[34] Arrieta Alejandro Barredo (2020). Explainable Artificial Intelligence (XAI): concepts, taxonomies, opportunities and challenges toward responsible AI. Inf. Fusion.

[35] ParsoonKottarathil (2020). PCOS dataset. https://www.kaggle.com/datasets/prasoonkottarathil/polycystic-ovary-syndrome-pcos.

[36] Guo Jiaqi (2024). Adaptive SV-Borderline SMOTE-SVM algorithm for imbalanced data classification. Appl. Soft Comput..

[37] Singh Dalwinder, Singh Birmohan (2020). Investigating the impact of data normalization on classification performance. Appl. Soft Comput..

[38] Theng Dipti, Bhoyar Kishor K. (2024). Feature selection techniques for machine learning: a survey of more than two decades of research. Knowl. Inf. Syst..

[39] Bharadiya J.P. (2023). A tutorial on principal component analysis for dimensionality reduction in machine learning. International Journal of Innovative Science and Research Technology.

[40] Castelli M., Manzoni L., Mariot L., Nobile M.S., Tangherloni A. (2022). Salp swarm optimization: a critical review. Expert Syst. Appl..

[41] Covert I.C., Qiu W., Lu M., Kim N.Y., White N.J., Lee S.I. (2023, July). International Conference on Machine Learning.

[42] Olivieri Alejandro C. (2024). Introduction to Multivariate Calibration: A Practical Approach.

[43] Jayachitra S. (2024). An efficient ranking based binary salp swarm optimization for feature selection in high dimensional datasets. Measurement: Sensors.

[44] Wei Bo (2024). Feature selection via a multi-swarm salp swarm algorithm. Electronic Research Archive.

[45] Zhou Hongfang, Wang Xiqian, Zhang Yao (2024). Feature selection based on weighted conditional mutual information. Appl. Comput. Inform..

[46] Francis Dallace, Sun Fengzhu (2024). A comparative analysis of mutual information methods for pairwise relationship detection in metagenomic data. BMC Bioinf..

[47] Liang Paul Pu, Zadeh Amir, Morency Louis-Philippe (2024). Foundations & trends in multimodal machine learning: principles, challenges, and open questions. ACM Comput. Surv..

[48] Van den Broeck Guy (2022). On the tractability of SHAP explanations. J. Artif. Intell. Res..

[49] Jabeur S.B., Mefteh-Wali S., Viviani J.L. (2024). Forecasting gold price with the XGBoost algorithm and SHAP interaction values. Ann. Oper. Res..

[50] Hajihosseinlou M., Maghsoudi A., Ghezelbash R. (2024). Stacking: a novel data-driven ensemble machine learning strategy for prediction and mapping of Pb-Zn prospectivity in Varcheh district, west Iran. Expert Syst. Appl..

[51] Rainio Oona, Teuho Jarmo, Klén Riku (2024). Evaluation metrics and statistical tests for machine learning. Sci. Rep..

[52] Chicco D., Jurman G. (2020). "The advantages of the Matthews correlation coefficient (MCC) over F1 score and accuracy in binary classification evaluation. BMC Genom..

[53] Kim J.J., Hwang K.R., Lee D., Kim S., Choi Y.M. (2024). Adolescents diagnosed with polycystic ovary syndrome under the Rotterdam criteria but not meeting the diagnosis under the updated guideline. Hum. Reprod..

[54] Unfer V., Kandaraki E., Pkhaladze L., Roseff S., Vazquez-Levin M.H., Laganà A.S., Nestler J. (2024). When one size does not fit all: reconsidering PCOS etiology, diagnosis, clinical subgroups, and subgroup-specific treatments. Endocrine and Metabolic Science.

[55] Wang Z., Jukic A.M.Z., Baird D.D., Wilcox A.J., Li H., Curry C.L., Mahalingaiah S. (2024). Irregular cycles, ovulatory disorders, and cardiometabolic conditions in a US-based digital cohort. JAMA Netw. Open.

[56] Benham J.L., Corbett K.S., Yamamoto J.M., McClurg C., Piltonen T., Yildiz B.O., Brown W.A. (2024). Impact of bariatric surgery on anthropometric, metabolic, and reproductive outcomes in polycystic ovary syndrome: a systematic review and meta‐analysis. Obes. Rev..

[57] Melo R.H., Pontes A.G., Delmanto L.R.M.G., Bueloni‐Dias F.N., Vespoli H.D.L., Nahas E.A.P. (2024). The role of glycated hemoglobin in the diagnosis of prediabetes and diabetes mellitus in young women with polycystic ovary syndrome. Clin. Endocrinol..

[58] Barbagallo F., van der Ham K., Willemsen S.P., Louwers Y.V., Laven J.S. (2024). Age-related curves of AMH using the gen II, the picoAMH, and the elecsys assays in women with polycystic ovary syndrome. The Journal of Clinical Endocrinology & Metabolism.

[59] Kamran H.S., Saeed T. (2024). Polycystic Ovary Syndrome.

[60] Medha S.L., Rickvibhadhini V., Sheetal B., Prajwala T.R. (2024, April). 2024 IEEE 9th International Conference for Convergence in Technology (I2CT).

[61] van der Ham K., Moolhuijsen L.M., Brewer K., Sisk R., Dunaif A., Laven J.S., Visser J.A. (2024). Clustering identifies subtypes with different phenotypic characteristics in women with polycystic ovary syndrome. The Journal of Clinical Endocrinology & Metabolism.

[62] Bokaie M., Khalesi Z.B., Farajkhoda T. (2024). Sexual and reproductive health concerns in women with polycystic ovary syndrome and their spouses: a qualitative study. Archives of Health Science and Research.

[63] Dinesh Paidipati, Vickram A.S., Kalyanasundaram P. (2024).

[64] UCI Machine Learning, Kaggle, “Breast Cancer Wisconsin (Diagnostic) Data Set”,https://www.kaggle.com/datasets/uciml/breast-cancer-wisconsin-data.

[65] Mansoor Iqbal, Kaggle, “Chronic KIdney Disease dataset”,https://www.kaggle.com/datasets/mansoordaku/ckdisease.

[66] Rahman Md Mustafizur, Al-Amin Md, Hossain Jahangir (2024). Machine learning models for chronic kidney disease diagnosis and prediction. Biomed. Signal Process Control.

[67] John Smith, Kaggle,https://www.kaggle.com/datasets/johnsmith88/heart-disease-dataset.

[68] Anjum Nishat (2024). Improving cardiovascular disease prediction through comparative analysis of machine learning models. Journal of Computer Science and Technology Studies.

[69] Yusuf Dede, Kaggle, https://www.kaggle.com/datasets/yusufdede/lung-cancer-dataset.

[70] Bhuiyan Mohammad Shafiquzzaman (2024). Advancements in early detection of lung cancer in public health: a comprehensive study utilizing machine learning algorithms and predictive models. Journal of Computer Science and Technology Studies.

[71] Munjal Ashok, Khandia Rekha, Gautam Brijraj (2020). A machine learning approach for selection of Polycystic Ovarian Syndrome (PCOS) attributes and comparing different classifier performance with the help of WEKA and PyCaret. Int. J. Sci. Res..

[72] Denny Amsy (2019). TENCON 2019-2019 IEEE Region 10 Conference (TENCON).

[73] Bharati Subrato, Podder Prajoy, Rubaiyat Hossain Mondal M. (2020). 2020 IEEE Region 10 Symposium (TENSYMP).

[74] Chitra P. (2022). 2022 4th International Conference on Inventive Research in Computing Applications (ICIRCA).

[75] Panda O.K., Sahoo S.S., Patra D., Rout T.R., Rakesh D.K., Behera B., Kumar R.R. (2024, March). 2024 1st International Conference on Cognitive, Green and Ubiquitous Computing (IC-CGU).

[76] Hdaib Dana (2022). 2022 5th International Conference on Engineering Technology and its Applications (IICETA).

[77] Tiwari Shamik (2022). SPOSDS: a smart Polycystic Ovary Syndrome diagnostic system using machine learning. Expert Syst. Appl..

[78] Rahman Md Mahbubur (2024). Empowering early detection: a web-based machine learning approach for PCOS prediction. Inform. Med. Unlocked.

[79] Suha Sayma Alam, Islam Muhammad Nazrul (2023). Exploring the dominant features and data-driven detection of polycystic ovary syndrome through modified stacking ensemble machine learning technique. Heliyon.

[80] Inan Muhammad Sakib Khan (2021). 2021 IEEE 11th Annual Computing and Communication Workshop and Conference (CCWC).

[81] Khanna Varada Vivek (2023). A distinctive explainable machine learning framework for detection of polycystic ovary syndrome. Applied System Innovation.

[82] Rahman Md Mahbubur (2022). A web-based heart disease prediction system using machine learning algorithms. Network Biology.

[83] Nagavelli Umarani, Samanta Debabrata, Partha Chakraborty (2022). Machine learning technology-based heart disease detection models. Journal of Healthcare Engineering.

[84] Das Akhil Kumar (2024). Machine learning based intelligent system for breast cancer prediction (MLISBCP). Expert Syst. Appl..

[85] Islam Md Ariful, Majumder Md Ziaul Hasan, Hussein Md Alomgeer (2023). Chronic kidney disease prediction based on machine learning algorithms. J. Pathol. Inf..

[86] Ibrahim Muhammad (2024). Fuzzy-based fusion model forβ-thalassemia carriers prediction using machine learning technique. Advances in Fuzzy Systems.

[87] Alatrany A.S., Khan W., Hussain A., Kolivand H., Al-Jumeily D. (2024). An explainable machine learning approach for Alzheimer's disease classification. Sci. Rep..

[88] DeGroat W., Abdelhalim H., Patel K., Mendhe D., Zeeshan S., Ahmed Z. (2024). Discovering biomarkers associated and predicting cardiovascular disease with high accuracy using a novel nexus of machine learning techniques for precision medicine. Sci. Rep..

[89] Hassoon A.R., Al-Naji A., Khalid G.A., Chahl J. (2024). Tongue disease prediction based on machine learning algorithms. Technologies.

[90] Laghmati S., Hamida S., Hicham K., Cherradi B., Tmiri A. (2024). An improved breast cancer disease prediction system using ML and PCA. Multimed. Tool. Appl..

[91] Reza M.S., Amin R., Yasmin R., Kulsum W., Ruhi S. (2024). Improving diabetes disease patients classification using stacking ensemble method with PIMA and local healthcare data. Heliyon.

